# The Elastic Critical Moment of Lateral Torsional Buckling of Steel Beams with Spatially Elastically Restrained at the Support Nodes

**DOI:** 10.3390/ma19010120

**Published:** 2025-12-29

**Authors:** Rafał Piotrowski, Andrzej Szychowski

**Affiliations:** Faculty of Civil Engineering and Architecture, Kielce University of Technology, Al. Tysiąclecia Państwa Polskiego 7, 25-314 Kielce, Poland; aszychow@tu.kielce.pl

**Keywords:** critical moment of lateral torsional buckling, elastic restraint against warping, elastic restraint against lateral rotation, elastic restraint against rotation in the beam bending plane, approximation formulas

## Abstract

This paper presents the results of a further stage of the authors’ research into the lateral torsional buckling of hot-rolled bisymmetric I-beams, spatially elastically restrained at the support nodes, i.e., against: (1) warping, (2) rotation in the lateral torsional buckling plane and (3) rotation in the main bending plane *M_y_*. The analysis considered the entire range of variation in node stiffness, from free support in bending *M_y_* and full freedom of warping and rotation in the lateral torsional buckling plane, to full restraint of the beam at the nodes. The authors introduced a general approximation formula (AF) for the critical moment of lateral torsional buckling *M_cr_*, simultaneously considering the three elastic fixity indexes for basic and frequently occurring loading schemes in engineering practice. In order to facilitate the calculations, the authors have included the full sequence of formulas for the successive components of the critical moment, derived in the authors’ previous papers. The ability to more accurately consider the actual conditions of the spatial elastic restraint of the beam at the nodes leads to a more accurate calculation of *M_cr_*. The results obtained were compared with FEM (LTBeamN software, v. 1.0.3) by performing a large number of calculations and numerical simulations. The agreement of the AF/FEM results was achieved at a level sufficient from the engineering point of view (mean value 1.006, standard deviation 0.028, coefficient of variation 2.8%). Detailed calculations were carried out for different section types (I, H) and different combinations of fixity index values. The application of approximation formulas in practical calculations is demonstrated on an example. The formulas derived in the paper can be used, among other things, to verify the correctness of FEM calculations, including the correct modelling of elastic support restraints, which is important in design practice.

## 1. Introduction

One of the important developments in modern methods of designing steel structures are the efforts to reproduce the behaviour of members and whole structures as accurately as possible in engineering computational models. For the design of members in bending, this includes consideration of the actual support conditions of beams sensitive to lateral torsional buckling. So far, the rather widely accepted scheme of the so-called ‘fork’ support has been inadequate in many technically significant cases. Such beams can be found, for example, in frame structures, flat or spatial frames and grillages.

The resistance of a member of such a structure depends both on the properties of the member under consideration and on the properties of its nodes and the properties of the other components of the system reaching these nodes. This is particularly true of so-called fully continuous systems and, to some extent, semi-continuous systems [1,2]. Only in so-called simple systems (nominally pinned connections at the nodes) the resistance of the beam is usually determined by the properties of the beam itself.

As has been shown in numerous theoretical studies, e.g., [3,4], experimental studies, e.g., [5,6], and numerical simulations, e.g., [3,7,8], the actual nodal boundary conditions, e.g., the support conditions of beams (members in bending) in, for instance, fully continuous structural systems, can be significantly different from the simplifications commonly adopted in design practice (e.g., the so-called ‘fork’ support model instead of elastic restraint). This affects the accuracy of the determination of the elastic critical load, in the case of beams—the critical moment of lateral torsional buckling (*M_cr_*). The elastic critical moment (*M_cr_*) is an important design parameter [1] which directly affects the so-called relative slenderness and indirectly—the reduction factor of the resistance of the beam in bending. The reduction factor (*χ_LT_*) takes into account both the phenomenon of member instability and unavoidable imperfections (geometric, residual stresses, etc.).

The consequence of the lateral-torsional buckling of the beam is the appearance of a complex load condition of the cross-section in the post-critical range. A beam exposed to bending in a single plane, loaded with the moment *M_y_* < *M_cr_* (i.e., in the elastic pre-critical range), after lateral torsional buckling, becomes a member bent in two planes (*M_y_*, *M_z_*) and is additionally in free (*M_v_*) and warping torsion (*M_ω_* + *B_ω_*). The moments of free *M_v_* and warping *M_ω_* torsion generate additional shear stresses, while the flexural-torsional bimoment *B_ω_* induces additional normal stresses, distributed in the cross-section according to the law of sectorial coordinates [9]. The complex stress state that arises in this case is the reason why the load-bearing capacity of the most stressed cross-section of the beam is exhausted much more quickly compared to members structurally protected against lateral torsional buckling.

Obviously, due to the unavoidable imperfections of the beams, the complex stress state, resulting from lateral-torsional deformations, is already present in the pre-critical range (*M_y_* < *M_cr_*). In order to consider the imperfect member model, it is necessary to determine the relative slenderness at lateral torsional buckling (${\bar{\;\lambda }}_{LT}$) and the reduction factor ($\chi_{LT}$) of the design resistance of the beam [1]. In view of the above, knowledge of *M_cr_* for the so-called perfect bar model and its loading is of considerable practical importance, since *M_cr_* is a parameter of the aforementioned slenderness, which, together with the imperfection parameter defined in the standard [1], enables the correct determination of factor $\chi_{LT}$.

The elastic critical resistance (ECR), which can be measured by the external critical load or critical moment *M_cr_* of a beam bending about the major axis of the cross-section, depends on: (1) the type and geometrical characteristics of the beam cross-section, e.g., [10,11,12], (2) nodal boundary conditions (mode of support and any elastic restraints present), e.g., [5,6], (3) the longitudinal distribution of the bending moment *M_y_*, e.g., [4,13,14], (4) the ordinate of the point of application of the transverse load relative to the shear centre of the cross-section, e.g., [15], (5) the possible presence of point-type and/or continuous bracing along the beam, e.g., [16,17], (6) the possible presence of an axial force, e.g., [18,19] and, (7) the effect of the pre-critical deflection of the beam in the bending plane on lateral torsional buckling, e.g., [16].

The analytical or approximation solutions currently known and used in design practice do not, in the general case, allow all the above-mentioned parameters affecting the *M_cr_* of the beam to be taken into account simultaneously. In the case of transversely bent beams, the methods of determining *M_cr_* for the theoretical ‘fork’ support in different load schemes are well recognised and described. For a more in-depth discussion of the extensive literature on the influence of selected parameters on the *M_cr_* of beams with ‘fork’ support, see, among others, the authors’ previous articles [20,21].

A separate group consists of works on the use of so-called ‘incomplete’ end plates [22,23,24] and methods of considering various details of coped beams [22,23,25,26,27,28,29,30] that weaken the way the beam is supported in comparison with the ‘fork’ support. In this case, a simplification of the computational model of nodal boundary conditions to a ‘fork’ support can lead to significant errors to the detriment of safety [22,23,24,25,26,27,28,29]. Correct consideration of such design cases is also extremely important from the point of view of the reliability of the entire structural system.

As already mentioned, there are also beam fixings (e.g., in the case of spandrel beams) in steel frame structures, particularly fully continuous structures, where the adoption of a ‘fork’ support scheme for critical moment calculations will be too conservative [7]. This concerns solutions for connections and nodes that allow for full or partial continuity in the transmission of cross-section forces, including bimoments, and displacements, including warping. These support conditions of the beam generate elastic restraint at the support nodes, which translates into an increase in the elastic critical moment.

In general, there may be a design case in which it is possible to very precisely consider, for instance, the effect of the ordinate of load application and/or the complex distribution of *M_y_* along the beam length, but all this effort is nullified by the adoption of over-simplified boundary conditions, which deviate from the working conditions of the actual member.

The effectiveness of the elastic restraint of a beam at a node depends on the structural design of the connection, the configuration of the additional stiffeners and the stiffness and degree of effort of the other beams reaching the node. Consideration of the full stiffness of the beams reaching the node is possible with sufficiently stiff (fully continuous) connections, e.g., welded joints [7].

In papers [3,31,32,33,34,35,36], for instance, the authors only consider the effect of elastic restraint against warping in beam support sections in the computational model. In [8], however, the authors consider the effect of elastic restraint against warping and twisting relative to the longitudinal axis of the beam (about the *x*–*x* axis).

In [3], an analytical formulation for the evaluation of the critical moment was proposed, taking into account the elastic restraint only against warping at the supports. Theoretically, the exact solution was derived from the differential equilibrium equations using infinite power series. However, the obtained solution is very complicated and not directly suitable for engineering calculations. The energy method for a reduced number of series terms was used to derive approximate expressions for the critical moment. Approximate formulas were obtained for loading with constant or linear distribution of bending moments and for transverse loads (i.e., concentrated force at the mid-span and uniformly distributed load) applied only to the shear center of the bisymmetric cross-section. The results were verified by FEM simulations.

The original application of the Rayleigh quotient method in the analysis of stability of straight elastic bars with arbitrary cross-sections was presented in [37]. The considerations take into account the so-called Vlasov-like theory, in which the cross-sectional characteristics and stress state components are calculated using formulas other than those in the classical Vlasov theory [9]. In the article [37] explicit forms of Rayleigh quotients relating to selected types of loads are given. The comparisons took into account the lateral torsional buckling of simply supported beams elastically restrained against warping on supports.

The technical literature lacks studies on lateral torsional buckling of beams whose elastic restraint at the support nodes goes beyond the restraint of warping or bending about the axis of greater stiffness [4]. This is due, among other things, to the considerable complexity of the problem, especially when attempting to consider elastic restraint conditions in the bending plane *M_y_*. In this case, there is a change in the location of the extreme moment *M_y,max_* (regardless of the sign of this moment), which corresponds to the engineering interpretation of the critical moment *M_cr_*. This is the result of a change in the stiffness of the elastic restraint of the support section on *M_y_*. For example, for a uniformly loaded beam simply supported in bending *M_y_*, the maximum bending moment *M_y,max_* = *ql*^2^/8 occurs at the midspan and causes compression of the top flange. However, in the case of a fully bilaterally fixed beam under the same load, *M_y,max_* = *ql*^2^/12 occurs on the support and causes maximum compression of the bottom flange. A detailed description of the phenomenon of the change in the location of the extreme value of the critical moment depending on the degree of elastic restraint is provided in the paper [21].

In previous papers [20,21,36,38], the authors determined the elastic critical moment *M_cr_* of the beam while considering the influence of different sets of elastic actions of the nodes. In paper [36], the authors considered the effect of elastic restraint against warping. In theoretical research, the original ‘coupling’ of the power polynomials describing the ‘deflection’ function of a simply supported beam and the ‘deflection’ function of a bilaterally fixed beam were used to approximate the twist angle function (*φ*). In the next stage of the research [20], the authors considered the interaction of elastic restraint against warping and elastic restraint of rotation in the lateral torsional buckling plane. In the theoretical research, the ‘coupling’ of polynomials proposed in [36] was applied to both the approximation of the twist angle function (*φ*) and the approximation of the lateral deflection function (*u*). *M_cr_* was determined using the energy method [39] in the *Rayleigh*-*Ritz* formulation. Based on symbolic calculations, approximation formulas for *M_cr_* were derived. A good approximation of *M_cr_* was obtained in comparison with FEM simulations using LTBeam. However, due to the use of two displacement functions *φ* and *u*, the obtained approximation formulas had a much more elaborate form than [36].

In the paper [21], in turn, the authors considered the interaction of elastic restraint against warping and elastic restraint of rotation in the bending plane *M_y_*. Elastic restraint against warping was considered in the same way as in the papers [20,36]. The effect of elastic restraint against rotation in the beam bending plane on the distribution of *M_y_* (which indirectly affects the value of *M_cr_*), in turn, was considered in the form of an appropriately calibrated interaction coefficient. This approach proved to be very successful even though the location of *M_y,max_* = *M_cr_* varied along the length of the beam, which directly depended on the stiffness of the elastic restraint against rotation about the major axis of the cross-section.

Consequently, in a subsequent paper [38], the authors extended this approach to the case of beams elastically restrained against warping and lateral rotation for the two extreme conditions of support for bending *M_y_* (i.e., pinned support and complete restraint). In addition to simplifying the formulas for *M_cr_*, this paper extends the results of the article [20] to the case of a beam completely restrained on *M_y_*.

The approximation solutions obtained in the papers [7,20,21,34,36,38] allow for the estimation of the elastic critical moments *M_cr_* for: (1) interaction of double configurations of elastic restraint [20,21,38], (2) beams with flat or closed stiffeners at support nodes [34,36], (3) beams being components of simple grillages [7] and, (4) beams of flat frames [21]. The results obtained from the approximation solutions were confirmed by FEM simulations in LTBeam (v. 1.0.11), LTBeamN (v. 1.0.3), Abaqus (v. 6.12) and ConSteel (ConSteel 15) software.

However, in the case of spatial frame structures, even more complex (spatial) boundary conditions may occur at the nodes of the component beams of such structures, concerning warping and rotation about the two principal axes of the cross-section (*y*–*y* and *z*–*z*). It is a question of simultaneously considering the elastic restraint against warping, the rotation in the lateral torsional buckling plane and the rotation in the beam’s main bending plane *M_y_*. To the authors’ knowledge, there is a lack of unambiguous analytical or approximate formulas for *M_cr_* in the literature that consider all three of the aforementioned elastic properties of the nodes simultaneously.

Obviously, the critical moment of lateral torsional buckling of the beam, considering the elastic action of the support nodes, can be determined using the finite element method (FEM). The available software, e.g., LTBeamN (v. 1.0.3) or ConSteel (ConSteel 15), based on thin-walled finite bar elements (7 degrees of freedom at the node), allow for the introduction of appropriate elastic stiffnesses to represent the behaviour of the node. More advanced 3D modelling can also be applied using shell (e.g., ConSteel 15, Abaqus/CAE 2017) or volumetric (e.g., Abaqus) finite elements. This enables a more precise representation of the individual details of the nodes. However, the ability to analytically validate the obtained FEM simulation results, especially with spatial modelling, improves the safety of the structure already at the design stage. This is especially true for less experienced designers who may make mistakes when creating an advanced numerical model, especially when modelling the behaviour of nodes (e.g., elastic restraints). This mutual assurance of the methods for determining the relevant design parameters is of both didactic and practical importance, allowing the obtained results to be verified in a relatively simple and rapid manner.

Of course, it could be argued that the approximated approach presented here (the use of approximation formulas) only applies to a few (in this case—three) variants of the longitudinal load distribution of the beam and three typical points of load application at the height of the cross-section (TF—top flange, CG—centre of gravity, BF—bottom flange). However, a computational test using approximation formulas for a simple load distribution and the defined elastic restraints, confirming the result of FEM simulations (e.g., according to LTBeamN) will allow for the confirmation of the correctness of the elastic restraint model. Once the support model is verified in this way, it will be possible to return to the FEM simulation by specifying the load diagram required in the design. Verifying the correctness of the load configuration and determining the *M_y_* distribution is much easier than verifying the elastic model of the nodes and can be done using simple software for linear static analysis or even manual calculations.

There is a current trend of theoretical research aimed at developing exact or approximate formulas for the verification of FEM calculations, e.g., [3,37,40,41,42,43].

Due to the lack of articles presenting exact, analytical or approximative solutions that take into account the influence of spatial elastic restraint in nodes on the critical moment of lateral torsional buckling of a steel beam, the authors attempted to obtain a technically useful solution to this problem.

This paper deals with the ECR analysis of the lateral torsional buckling condition of a hot-rolled bisymmetric I-beam, assuming its spatial elastic restraint at the support nodes, e.g., in a spatial frame structure. In the presented computational model, three nodal parameters (i.e., elastic restraints) affecting the critical moment of lateral torsional buckling *M_cr_* were considered. The analysis considered the simultaneous occurrence of the following elastic support restraints: (1) against warping, (2) against lateral rotation (in the lateral torsional buckling plane) and (3) against rotation in the plane of bending *M_y_* (i.e., about the stronger axis of the cross-section). A full and mutually independent (as in LTBeamN) range of variation in the degree of elastic restraint, from simple support for bending *M_y_* and ‘fork’ support for lateral torsional buckling to full restraint at the nodes has been considered for the above-mentioned parameters.

The following assumptions were made in the work: (1) single-span beams have a prismatic, bisymmetric hot-rolled I-section or its welded equivalent, (2) the same boundary conditions are present at both nodes, (3) three most typical load scheme, most frequently encountered in engineering practice, are considered, (4) the conditions of the elastic restraint of the beam in the support sections consider two rotational degrees of freedom (about the principal axes of the cross-section) and one warping degree of freedom.

Compared to the solutions available in the literature, this paper offers a novel approach that takes into account:

(i) The derivation of the approximation formula for *M_cr_* while considering any degree of elastic restraint of node stiffness against: (a) warping, (b) rotation about the minor axis of the cross-section and, (c) rotation about the major axis of the cross-section (in the bending plane *M_y_*).
(ii) Obtaining an approximation solution that makes it possible to fairly easily consider the real behaviour of a steel beam sensitive to lateral torsional buckling that is incorporated in a fully continuous spatial frame structure.
(iii) The obtained formulas can be used, among other things, to verify the modelling of elastic support restraints in FEM calculations, for the load schemes most commonly found in practice and three variants of load application at the height of the cross-section. Once the node model has been validated in this way, a numerical solution for virtually any loading mode (e.g., according to LTBeamN) can be obtained in further FEM simulations.

## 2. Conditions for Spatial Elastic Restraint of the Beam at the Support Nodes

**Figure 1:**
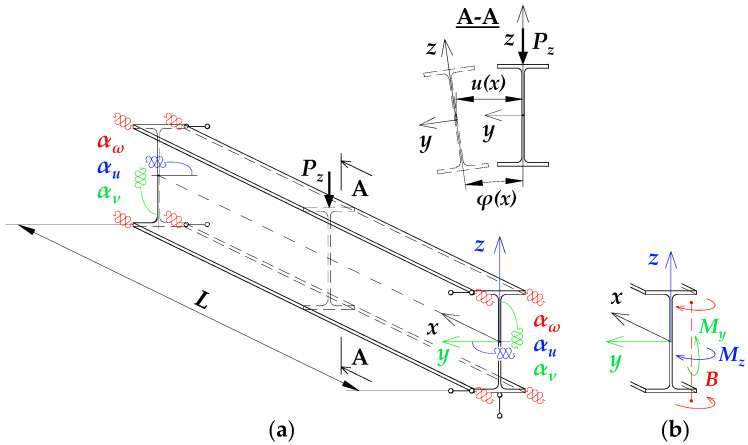
Static diagram of the beam: (**a**) restraint of warping ($\alpha_{\omega }$), restraint of lateral rotation ($\alpha_{u}$), restraint of rotation in the beam bending plane ($\alpha_{\nu }$), lateral deflection *u*(*x*), twist angle *φ*(*x*), (**b**) bimoment *B*, moment *M_z_*, moment *M_y_*.

A static scheme of a beam spatially elastically restrained at the support nodes is shown in Figure 1. In the analysis, the authors considered the stiffnesses of the elastic restraint of the node against: (1) warping $\alpha_{\omega }$ (red), (2) rotation in the lateral torsional buckling plane $\alpha_{u}$ (blue) and (3) rotation in the bending plane $\alpha_{\nu }$ (green).

Stiffnesses of elastic restraint $\alpha_{\omega }$ [20,21,35,36,44], $\alpha_{u}$ [20] and $\alpha_{\nu }$ [21] express the values of generalised internal forces (i.e., bimoment *B*, moment *M_z_* and moment *M_y_*) induced by unit generalised displacements (warping *dφ*/*dx*, lateral rotation *du*/*dx* and rotation in the bending plane *dv*/*dx*, respectively) in the support section of the beam. For example, the stiffness of elastic restraint against warping $\alpha_{\omega }$ is the value of the bimoment (*B*) induced by the unit warping of the support section (*dφ*/*dx*). Stiffnesses ($\alpha_{\omega }$, $\alpha_{u}$) concerning the so-called lateral torsional buckling boundary condition and the stiffness ($\alpha_{\nu }$) concerning the static boundary condition at beam supports vary from $\alpha_{\omega }=0$, $\alpha_{u}=0$ for a ‘fork’ support and $\alpha_{\nu }=0$ for the simple support on *M_y_*, to $\alpha_{\omega }=\infty$, $\alpha_{u}=\infty$ and $\alpha_{\nu }=\infty$ for complete restraint.

The elastic restraint, the same at the two support nodes, was considered via dimensionless fixity indexes $\kappa_{\omega }$ [20,21,36], $\kappa_{u}$ [20] and $\kappa_{\nu }$ [21].

The dimensionless index of elastic fixity against warping $\kappa_{\omega }$ was determined from $\alpha_{\omega }$ in the form [20,21,36]:

(1) $$\kappa_{\omega }=\frac{\alpha_{\omega }L}{2EI_{\omega }+\alpha_{\omega }L}\;,$$

where L—beam span, E—Young’s modulus, $I_{\omega }$—warping constant, and $\alpha_{\omega }$—rigidity of elastic restraint against warping [20,21,35,36,44].

The index of elastic fixity against warping changes is from $\kappa_{\omega }=0$ for complete warping freedom to $\kappa_{\omega }=1$ for full prevention of warping.

The dimensionless index of elastic fixity against lateral rotation (i.e., in the lateral torsional buckling plane) $\kappa_{u}$ was determined from $\alpha_{u}$ in the form [20]:

(2) $$\kappa_{u}=\frac{\alpha_{u}L}{2EI_{z}+\alpha_{u}L}\;,$$

where $I_{z}$—second moment of inertia in bending about the *z*-axis, and $\alpha_{u}$—rigidity of elastic restraint against lateral rotation [20].

The index of elastic fixity against lateral rotation changes is from $\kappa_{u}=0$ for complete lateral rotation freedom to $\kappa_{u}=1$ for full prevention of lateral rotation.

The dimensionless index of elastic fixity against rotation in the bending plane $\kappa_{\nu }$ was determined from $\alpha_{\nu }$ in the form [21]:

(3) $$\kappa_{\nu }=\frac{\alpha_{\nu }L}{4EI_{y}+\alpha_{\nu }L}\;,$$

where $I_{y}$—second moment of inertia in bending about the *y*-axis, $\alpha_{\nu }$—rigidity of elastic restraint against rotation in the beam bending plane [21].

The index of elastic fixity against rotation in the bending plane of the beam changes is from $\kappa_{\nu }=0$ for complete freedom of rotation (hinge support) to $\kappa_{\nu }=1$ for full prevention of rotation (fixity).

A simple transformation of Formulas (1)–(3) makes it possible to express stiffnesses ($\alpha_{\omega }$, $\alpha_{u}$, $\alpha_{\nu }$) as a function of the index ($\kappa_{\omega }$, $\kappa_{u}$, $\kappa_{\nu }$) of elastic restraint according to the formulas:

(4) $$\alpha_{\omega }=\frac{2\kappa_{\omega }EI_{\omega }}{\left(1-\kappa_{\omega }\right)L},\;\;\;\;\alpha_{u}=\frac{2\kappa_{u}EI_{z}}{\left(1-\kappa_{u}\right)L},\;\;\;\;\alpha_{\nu }=\frac{4\kappa_{\nu }EI_{y}}{\left(1-\kappa_{\nu }\right)L}.$$

## 3. Critical Moment of Lateral Torsional Buckling of Beams Spatially Elastically Restrained at the Support Nodes—$M_{cr}\left(\kappa_{\omega },\kappa_{u},\kappa_{v}\right)$

### 3.1. Examples of FEM Simulations According to LTBeamN

The development of the approximation formula was preceded, as in the paper [21], by an analysis of the variation of the critical moment as a function of elastic fixity indexes ($\kappa_{\omega }$, $\kappa_{u}$, $\kappa_{\nu }$) for a large set of technically relevant cases. In this section, in analysing the variation of $M_{cr}\left(\kappa_{v}\right)$, the authors considered the impact of imposed values $\kappa_{\omega }$ and $\kappa_{u}$.

**Figure 2:**
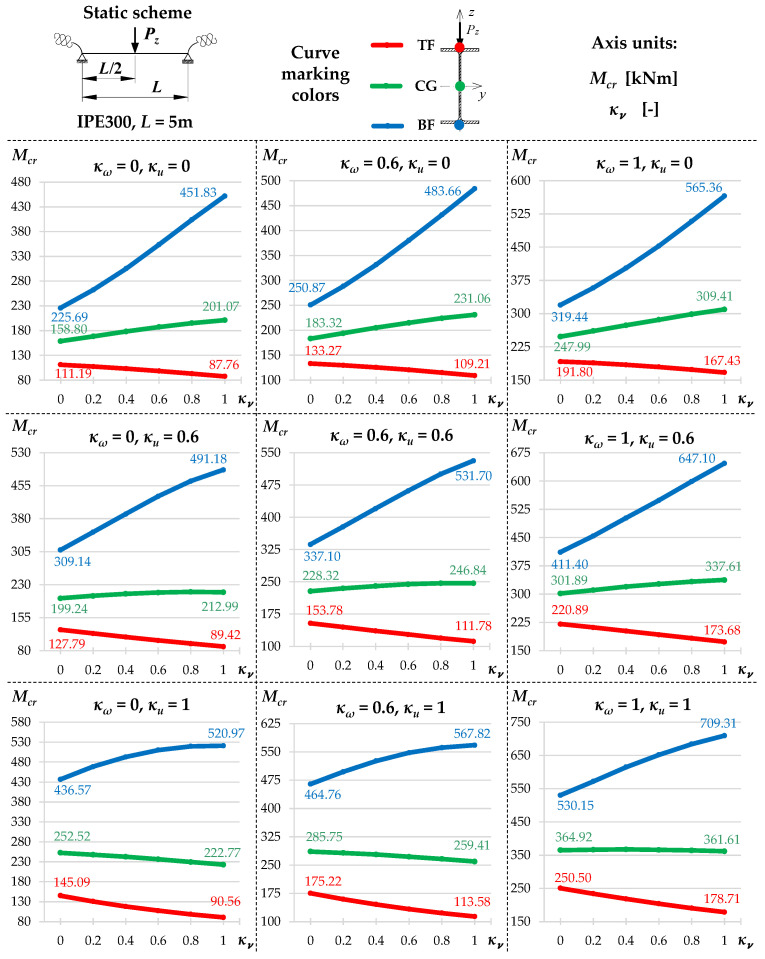
Trends in *M_cr_* variation according to LTBeamN software (v. 1.0.3) as a function of the fixity index $\kappa_{\nu }$ for beam under the concentrated force load (CFL) at the midspan.

**Figure 3:**
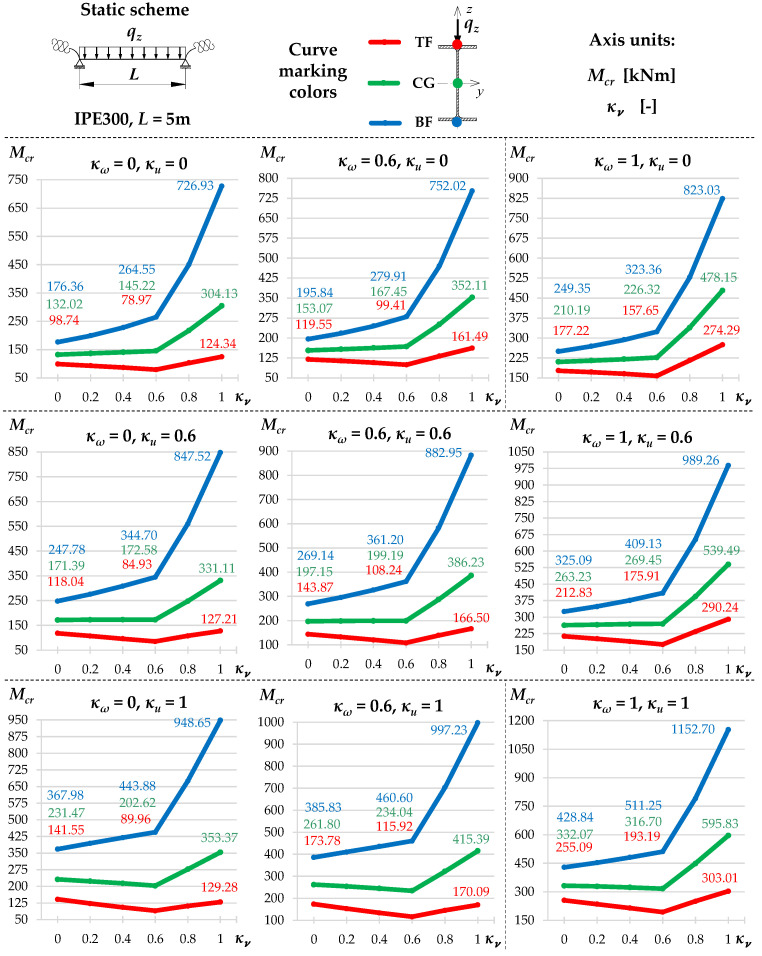
Trends in *M_cr_* variation according to LTBeamN software (v. 1.0.3) as a function of the fixity index $\kappa_{\nu }$ for beam under the uniform distributed load (UDL).

Figure 2 and Figure 3 show the variation of $M_{cr}\left(\kappa_{v}\right)$ for an IPE300 beam with the span *L* = 5 m, loaded with: (1) a concentrated load at midspan (CFL)—Figure 2, and (2) uniformly distributed load (UDL)—Figure 3, for selected values of indexes: $\kappa_{\omega }$ = {0, 0.6, 1} and $\kappa_{u}$ = {0, 0.6, 1}. The beam loads (CFL, UDL) were set at the height of the top flange (TF), the centre of gravity (CG) or the bottom flange (BF). The calculations were performed in the LTBeamN (FEM) software (v. 1.0.3).

The charts (Figure 2 and Figure 3) indicate that:

(1) The highest *M_cr_* was obtained for loads applied to the bottom flange (BF—blue line) and the lowest—for loads applied to the top flange (TF—red line). The load applied to the centre of gravity (CG—green line) produced intermediate *M_cr_* values.
(2) In all load cases, as the indexes $\kappa_{\omega }$ and $\kappa_{u}$ increased, for a given value of $\kappa_{\nu }$, *M_cr_* increased.
(3) The influence of the index $\kappa_{v}$ on *M_cr_* is more complicated and depends on the longitudinal distribution of the bending moment *M_y_* as a function of $\kappa_{v}$, as described in the following sections.
(4) In the case of a concentrated force (Figure 2) applied to the top flange (TF) and in some cases of loads applied to the centre of gravity (CG), there was a decrease in *M_cr_* across the entire range of variation of the index $0<\kappa_{\nu }\leq 1$. This is due to the change in the longitudinal distribution of *M_y_*, which is directly dependent on the value of the index $\kappa_{v}$. However, as the $\kappa_{v}$ increased (despite a decrease in the *M_cr_* value) the critical load *P_z,cr_* of the beam increased as well. This phenomenon is described in detail in the paper [21].
(5) For a uniformly distributed load (Figure 3), regardless of where it was applied (TF, CG, BF), there is a typical bend in the curve $M_{cr}\left(\kappa_{v}\right)$ at $\kappa_{v}=0.6$. In this case, the absolute values of the span and support moments are equal to each other. For $\kappa_{v}<0.6$, the maximum moment *M_y,max_* occurs in the span, and for $\kappa_{v}>0.6$, the maximum moment *M_y,max_* occurs above the beam support [21]. For a load applied to the top flange (TF—red line) and, in some cases (e.g., for $\kappa_{u}=1$), for a load applied to the centre of gravity (CG—green line), in the range: $0<\kappa_{\nu }\leq 0.6$, there is also a decrease in *M_cr_* values. This is due to the change in the location of *M_y,max_* (identified with *M_cr_*) as a function of $\kappa_{v}$. In this range of variation ($0<\kappa_{\nu }\leq 0.6$), as the value of $\kappa_{v}$ increases (despite a decrease in the *M_cr_* value), the critical load *q_z,cr_* of the beam also increases [21].

The successive cases of $M_{cr}\left(\kappa_{v}\right)$ variation examined in this work for different combinations of index values: (1) elastic restraint against warping: $\kappa_{\omega }$ = {0, 0.2, 0.4, 0.6, 0.8, 1} and (2) elastic restraint against lateral rotation: $\kappa_{u}$ = {0, 0.2, 0.4, 0.6, 0.8, 1} were similar to those shown in Figure 2 and Figure 3.

### 3.2. Approximation Formula for $M_{cr}\left(\kappa_{\omega },\kappa_{u},\kappa_{v}\right)$

**Figure 4:**
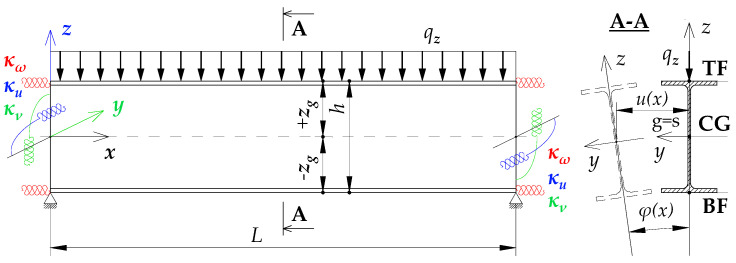
Static scheme of a beam elastically restrained in the support nodes ($\kappa_{\omega }$, $\kappa_{u}$, $\kappa_{v}$).

The approximation formula for the critical moment of lateral torsional buckling of the beam, spatially elastically restrained at the support nodes $M_{cr}\left(\kappa_{\omega },\kappa_{u},\kappa_{v}\right)$ (Figure 4), can be presented in the Formula (5), i.e., similar to that presented in the paper [21]. In this case, the components of Formula (5) were expanded to include the effect of the index $\kappa_{u}$, which accounts for the elastic restraint against lateral rotation in the lateral torsional buckling plane:

(5) $$\begin{array}{cc} & M_{cr}\left(\kappa_{\omega },\kappa_{u},\kappa_{v}\right)=M_{cr,o}\left(\kappa_{\omega },\kappa_{u},\kappa_{v}=0\right)\\ & \;\;\;\;\;\;+\left[M_{cr,u}\left(\kappa_{\omega },\kappa_{u}{,\kappa }_{v}=1\right)-M_{cr,o}\left(\kappa_{\omega },\kappa_{u}{,\kappa }_{v}=0\right)\right]\cdot \eta \left(\kappa_{v}\right)\;, \end{array}$$

where $M_{cr,o}\left(\kappa_{\omega },\kappa_{u},\kappa_{v}=0\right)$—the LTB critical moment according Formula (7) for a simply supported for *M_y_* beam and a given value of the $\kappa_{\omega }$ and $\kappa_{u}$ index, $M_{cr,u}\left(\kappa_{\omega },\kappa_{u}{,\kappa }_{v}=1\right)$—the LTB critical moment according Formula (10) for a bilaterally fixed for *M_y_* beam and a given value of the $\kappa_{\omega }$ and $\kappa_{u}$ index, $\eta \left(\kappa_{v}\right)$—the coefficient of interaction.

As already mentioned, in the paper [21], the derivation of approximation formulas for the interaction coefficient $\eta \left(\kappa_{v}\right)$ was preceded by numerical simulations (LTBeamN) of extensive sets of cases of single-span beams with hot-rolled sections (IPE, HEA and HEB). On this basis, it was concluded that in the case of: (1) a concentrated force load at midspan (CFL), irrespective of the point of load application, (2) uniformly distributed load (UDL) applied to the top flange (TF) and (3) triangular distributed load (TDL) (non-uniform load) applied to the top flange (TF), the critical load ($P_{cr}$, $q_{cr}$, $q_{Tcr}$) could be determined as a linear combination of the critical load for a beam simply supported in bending *M_y_* ($P_{o}$, $q_{o}$, $q_{To}$) and fully restrained in bending *M_y_* ($P_{u}$, $q_{u}$, $q_{Tu}$) according to the formulas (6) [21]:

(6) $$P_{cr}=\left(1-\kappa_{v}\right)P_{o}+\kappa_{v}P_{u}\;,\;q_{cr}=\left(1-\kappa_{v}\right)q_{o}+\kappa_{v}q_{u}\;,\;q_{Tcr}=\left(1-\kappa_{v}\right)q_{To}+\kappa_{v}q_{Tu}\;.$$

The tests performed in this research confirmed that for the above-mentioned load cases and for the additional consideration of the elastic restraint of the beam in the lateral torsional buckling plane ($0<\kappa_{u}\leq 1$), Equation (6) adequately describe the behaviour of the beams in the elastic critical condition, which means that the formulas for $\eta \left(\kappa_{v}\right)$ can be kept as in the paper [21].

On the other hand, for a uniformly or non-uniformly distributed load applied to the centre of gravity (CG) or the bottom flange (BF), the application of the coefficients determined in [21] (for $\kappa_{u}=0$) yielded overestimated *M_cr_* values compared to FEM (LTBeamN). Therefore, in the present research, for these two static schemes (UDL and TDL) and the loads applied to CG and BF, an additional calibration of the $\eta \left(\kappa_{v}\right)$ coefficients was performed.

In the paper [21], the interaction coefficients $\eta \left(\kappa_{v}\right)$ were calibrated using several sets of sections and their spans (IPE300, HEA300, HEB300 with spans *L* = 5 and 7 m and IPE500, HEA500, HEB500 with spans *L* = 8 and 10 m). On the basis of that research, it was concluded that sufficiently correct calibration results could be obtained from a detailed analysis of the results for an IPE300 beam with a span of *L* = 5 m. In view of the above, a different strategy was adopted in the paper [38]. The calibration of the interaction coefficient, in this case $\eta \left(\kappa_{u}\right)$, was carried out for an IPE300 beam for *L* = 5 m while extending the set of different section families (IPE, HEA, HEB) and their spans *L* to test for differences in *M_cr_* values. This approach significantly simplified the calculations while achieving an acceptable level of accuracy.

Therefore, in the present paper, the authors calibrated the remaining coefficients $\eta \left(\kappa_{v}\right)$ (i.e., for UDL and TDL loads applied to CG and BF) using the same method, i.e., detailed calculations were performed for an IPE300 beam with a span of *L* = 5 m. The authors considered the fixity indexes: $\kappa_{v}$ = {0, 0.2, 0.4, 0.6, 0.8, 1}, for uniform loading, and: $\kappa_{v}$ = {0, 0.2, 0.4, 0.564, 0.8, 1}, for non-uniform loading. The obtained results were tested for an extended range of sections from families (IPE, HEA, HEB). The results of the comparisons are presented in Section 6.

Finally, the formulas for the interaction coefficient $\eta \left(\kappa_{v}\right)$ adopted in this paper for spatially elastically restrained ($\kappa_{\omega }$, $\kappa_{u}$, $\kappa_{v}$) beams are given in Table 1.

Note: In the calculations of the critical moment of spatially elastically restrained beams $M_{cr}\left(\kappa_{\omega },\kappa_{u},\kappa_{v}\right)$ using the approximation Formula (5) and the integrated interaction coefficient according to Table 1, it should be assumed that the calculations are carried out for the $\kappa_{v}$ range of 0.1 to 0.9. At the extremes, i.e., for $\kappa_{v}$ from 0 to 0.1 and 0.9 to 1 (to improve accuracy), the values of *M_cr_* may be interpolated linearly, i.e., between the value of $M_{cr,o}\left(\kappa_{\omega },\kappa_{u},\kappa_{v}=0\right)$ from the Formula (7) and $M_{cr}\left(\kappa_{\omega },\kappa_{u},\kappa_{v}=0.1\right)$ from the Formula (5) for the lower interval (0 to 0.1), and between the value of $M_{cr}\left(\kappa_{\omega },\kappa_{u},\kappa_{v}=0.9\right)$ from the Formula (5) and $M_{cr,u}\left(\kappa_{\omega },\kappa_{u},\kappa_{v}=1\right)$ from the Formula (10) for the upper interval (from 0.9 to 1). This assumption made it possible to simplify the function of the interaction coefficient $\eta \left(\kappa_{v}\right)$, whose form for the full interval $\kappa_{v}$ (from 0 to 1) became excessively complicated.

In order to simplify practical calculations, in the next section of this article, the authors included approximation formulas and corresponding coefficients for determining the individual components $M_{cr}\left(\kappa_{\omega },\kappa_{u}\right)$ needed for the final determination of $M_{cr}\left(\kappa_{\omega },\kappa_{u},\kappa_{v}\right)$. The formulas were derived from earlier work by the authors [21,36,38].

## 4. Calculation of the Components $M_{cr}\left(\kappa_{\omega },\kappa_{u}\right)$ for the Formula (5)

### 4.1. $M_{cr,o}\left(\kappa_{\omega },\kappa_{u},\kappa_{v}=0\right)$—for a Beam Simply Supported in the Bending Plane M_y_ ($\kappa_{v}=0$) and Any Values of the Indexes $\kappa_{\omega }$ and $\kappa_{u}$

$M_{cr,o}\left(\kappa_{\omega },\kappa_{u},\kappa_{v}=0\right)$ can be calculated using the Formula (7), which is a version of the formula proposed in the paper [38] written with the indicator $\kappa_{v}$. After considering the notations related to the freedom of support of the beam in its bending plane *M_y_* ($\kappa_{v}=0$), the formula has the form:

(7) $$M_{cr,o}\left(\kappa_{\omega },\kappa_{u},\kappa_{v}=0\right)=\;\;\;\;\;\;\;\;\;\;\;\;\;\;\;=M_{cr}\left(\kappa_{\omega },\kappa_{u}=0,\kappa_{v}=0\right)\;\;\;\;\;\;\;\;\;\;\;\;\;\;\;+\left[M_{cr}\left(\kappa_{\omega },\kappa_{u}=1,\kappa_{v}=0\right)-M_{cr}\left(\kappa_{\omega },\kappa_{u}=0,\kappa_{v}=0\right)\right]\cdot \eta_{o}\left(\kappa_{u}\right)\;,$$

where $M_{cr}\left(\kappa_{\omega },\kappa_{u}=0,\kappa_{v}=0\right)$—the LTB critical moment for a simply supported for *M_y_* beam with complete freedom of lateral rotation ($\kappa_{u}=0$) and a given value of the $\kappa_{\omega }$ index, $M_{cr}\left(\kappa_{\omega },\kappa_{u}=1,\kappa_{v}=0\right)$—the LTB critical moment for a simply supported for *M_y_* beam with complete blockage of lateral rotation ($\kappa_{u}=1$) and a given value of the $\kappa_{\omega }$ index, $\eta_{o}\left(\kappa_{u}\right)$—the coefficient of interaction (for $\kappa_{v}=0$) [38].

Formulas for individual components of the ‘lower order’ $M_{cr}\left(\kappa_{\omega },\kappa_{u}=0,\kappa_{v}=0\right)$ and $M_{cr}\left(\kappa_{\omega },\kappa_{u}=1,\kappa_{v}=0\right)$ and the interaction coefficient $\eta_{o}\left(\kappa_{u}\right)$ are provided in Section 4.1.1, Section 4.1.2 and Section 4.1.3.

Note: Another, more elaborate, form of the approximation formula for $M_{cr,o}\left(\kappa_{\omega },\kappa_{u},\kappa_{v}=0\right)$ is given in the paper [20]. The solution obtained there relates to a beam simply supported against bending *M_y_*, considering elastic restraint against warping $\kappa_{\omega }$ and rotation about the minor axis of the section $\kappa_{u}$. A discussion of the differences in the solution according to Formula (7) and the corresponding formula from the paper [20] has been included in [38].

#### 4.1.1. $M_{cr}\left(\kappa_{\omega },\kappa_{u}=0,\kappa_{v}=0\right)$—for a Beam Simply Supported in the Lateral Torsional Buckling Plane ($\kappa_{u}=0$) and Any Values of the Index $\kappa_{\omega }$

For a beam simply supported ($\kappa_{v}=0$) in the bending plane, unrestrained against rotation about the minor axis of the section ($\kappa_{u}=0$) and elastically restrained against warping ($0\leq \kappa_{\omega }\leq 1$), the critical moment of lateral torsional buckling can be determined using the formula given in [36]:

(8) $$M_{cr}\left(\kappa_{\omega },\kappa_{u}=0,\kappa_{v}=0\right)=\frac{-B_{1}EI_{z}z_{g}+\sqrt{EI_{z}\left(B_{3}GI_{t}L^{2}+B_{4}EI_{\omega }+B_{1}^{2}EI_{z}z_{g}^{2}\right)}}{B_{2}L^{2}}\;,$$

where $B_{1},\;B_{2},\;B_{3},\;B_{4}$—coefficients according to Table 2, $z_{g}$—ordinate of the point of transverse load application with respect to shear centre (see Figure 4), h—high of the beam (see Figure 4).

Table 2 lists the $B_{1},\;B_{2},\;B_{3}$ and $B_{4}$ coefficients for beams simply supported in bending ($\kappa_{v}=0$), with complete freedom of lateral rotation ($\kappa_{u}=0$) and the most common loading schemes [36].

#### 4.1.2. $M_{cr}\left(\kappa_{\omega },\kappa_{u}=1,\kappa_{v}=0\right)$—For a Beam Fully Fixed in the Lateral Torsional Buckling Plane ($\kappa_{u}=1$) and Any Values of the Index $\kappa_{\omega }$

For a beam simply supported ($\kappa_{v}=0$) in the bending plane, with full restraint against lateral rotation ($\kappa_{u}=1$) and elastic restraint against warping ($0\leq \kappa_{\omega }\leq 1$), the critical moment of lateral torsional buckling can be determined using the formula given in [38]:

(9) $$M_{cr}\left(\kappa_{\omega },\kappa_{u}=1,\kappa_{v}=0\right)=D_{1}\frac{-B_{1}EI_{z}z_{g}+\sqrt{EI_{z}\left(B_{3}GI_{t}L^{2}+B_{4}EI_{\omega }+B_{1}^{2}EI_{z}z_{g}^{2}\right)}}{B_{2}L^{2}}\;,$$

where $B_{1},\;B_{2},\;B_{3},\;B_{4},\;D_{1}$—coefficients according to Table 3, $z_{g}$—ordinate of the point of transverse load application with respect to shear centre (see Figure 4), h—high of the beam (see Figure 4).

Table 3 lists the $B_{1},\;B_{2},\;B_{3},\;B_{4}$ and $D_{1}$ coefficients for beams simply supported in bending ($\kappa_{v}=0$), with complete blockage of lateral rotation ($\kappa_{u}=1$) and the most common loading schemes [38].

#### 4.1.3. Interaction Coefficient $\eta_{o}\left(\kappa_{u}\right)$

For a beam simply supported in bending *M_y_* ($\kappa_{v}=0$), elastically restrained at the support nodes against warping ($0\leq \kappa_{\omega }\leq 1$) and lateral rotation ($0<\kappa_{u}<1$), the interaction coefficient $\eta_{o}\left(\kappa_{u}\right)$ has been determined in the paper [38] and shown in Table 4.

### 4.2. $M_{cr,u}\left(\kappa_{\omega },\kappa_{u}{,\kappa }_{v}=1\right)$—For a Beam Fully Fixed in the Bending Plane M_y_$\left(\kappa_{v}=1\right)$ and Any Values of the Indexes $\kappa_{\omega }$ and $\kappa_{u}$

$M_{cr,u}\left(\kappa_{\omega },\kappa_{u}{,\kappa }_{v}=1\right)$ can be calculated using the Formula (7), which is a version of the formula proposed in the paper [38] written with the indicator $\kappa_{v}$. After considering the notations related to the full restraint of the beam in its bending plane *M_y_* ($\kappa_{v}=1$), the formula has the form:

(10) $$M_{cr,u}\left(\kappa_{\omega },\kappa_{u},\kappa_{v}=1\right)=M_{cr}\left(\kappa_{\omega },\kappa_{u}=0,\kappa_{v}=1\right)+\left[M_{cr}\left(\kappa_{\omega },\kappa_{u}=1,\kappa_{v}=1\right)-\;M_{cr}\left(\kappa_{\omega },\kappa_{u}=0,\kappa_{v}=1\right)\right]\cdot \eta_{u}\left(\kappa_{u}\right),$$

where $M_{cr}\left(\kappa_{\omega },\kappa_{u}=0,{\;\kappa }_{v}=1\right)$—the LTB critical moment for a bilaterally fixed for *M_y_* beam with complete freedom of lateral rotation ($\kappa_{u}=0$) and a given value of the $\kappa_{\omega }$ index, $M_{cr}\left(\kappa_{\omega },\kappa_{u}=1,\kappa_{v}=1\right)$—the LTB critical moment for a bilaterally fixed for *M_y_* beam with complete blockage of lateral rotation ($\kappa_{u}=1$) and a given value of the $\kappa_{\omega }$ index, $\eta_{u}\left(\kappa_{u}\right)$—the coefficient of interaction (for $\kappa_{v}=1$) [38].

Formulas for individual components of the ‘lower order’ $M_{cr}\left(\kappa_{\omega },\kappa_{u}=0,\kappa_{v}=1\right)$ and $M_{cr}\left(\kappa_{\omega },\kappa_{u}=1,\kappa_{v}=1\right)$ and the interaction coefficient $\eta_{u}\left(\kappa_{u}\right)$ are provided in Section 4.2.1, Section 4.2.2 and Section 4.2.3.

#### 4.2.1. $M_{cr}\left(\kappa_{\omega },\kappa_{u}=0,\kappa_{v}=1\right)$—For a Beam Simply Supported in the Lateral Torsional Buckling Plane ($\kappa_{u}=0$) and Any Values of the Index $\kappa_{\omega }$

The critical moment $M_{cr}\left(\kappa_{\omega },\kappa_{u}=0,\kappa_{v}=1\right)$ of lateral torsional buckling of the beam fully fixed in the bending plane *M_y_* ($\kappa_{v}=1$), considering the full freedom of lateral rotation ($\kappa_{u}=0$) and elastic restraint against warping ($0\leq \kappa_{\omega }\leq 1$), can be determined using the Formula (8) [36]. In this case, the formulas for the coefficients $B_{1},\;B_{2},\;B_{3},\;B_{4}$ are given in Table 5 [21].

#### 4.2.2. $M_{cr}\left(\kappa_{\omega },\kappa_{u}=1,\kappa_{v}=1\right)$—For a Beam Fully Fixed in the Lateral Torsional Buckling Plane ($\kappa_{u}=1$) and Any Values of the Index $\kappa_{\omega }$

The critical moment $M_{cr}\left(\kappa_{\omega },\kappa_{u}=1,\kappa_{v}=1\right)$ of lateral torsional buckling of the beam fully fixed in the bending plane *M_y_* ($\kappa_{v}=1$), considering the full restraint of lateral rotation ($\kappa_{u}=1$) and elastic restraint against warping ($0\leq \kappa_{\omega }\leq 1$), can be determined using the Formula (9) specified in [38]. In this case, the formulas for the coefficients $B_{1},\;B_{2},\;B_{3},\;B_{4},\;D_{1}$ are given in Table 6 [38].

#### 4.2.3. Interaction Coefficient $\eta_{u}\left(\kappa_{u}\right)$

For a beam fully fixed against *M_y_* ($\kappa_{v}=1$), elastically restrained at the support nodes against warping ($0\leq \kappa_{\omega }\leq 1$) and lateral rotation ($0<\kappa_{u}<1$), the interaction coefficient has been determined in the paper [38] and shown in Table 7.

## 5. Calculation Procedure $M_{cr}\left(\kappa_{\omega },\kappa_{u},\kappa_{v}\right)$

In order to unambiguously present the calculation sequence for determining the critical moment $M_{cr}\left(\kappa_{\omega },\kappa_{u},\kappa_{v}\right)$ of a spatially elastically restrained beam, this authors describe the relevant procedure in this section.

Sequence of calculations:

(1) define basic design parameters of the beam: cross-section (I, H), span (*L*), type of loading (CFL, UDL, TDL);
(2) based on the design case (support conditions of the beam), determine the stiffness of the elastic restraints against: warping $\alpha_{\omega }$, lateral rotation (in the lateral torsional buckling plane) $\alpha_{u}$ and rotation in the plane of bending $\alpha_{\nu }$;
(3) determine elastic fixity indexes $\kappa_{\omega }$, $\kappa_{u}$ and $\kappa_{\nu }$ using the Formulas (1)–(3);
(4) for a given $\kappa_{\omega }$, determine $M_{cr}\left(\kappa_{\omega },\kappa_{u}=0,\kappa_{v}=0\right)$ using the Formula (8) and $M_{cr}\left(\kappa_{\omega },\kappa_{u}=1,\kappa_{v}=0\right)$ the Formula (9);
(5) for a given $\kappa_{u}$, determine the interaction coefficient $\eta_{o}\left(\kappa_{u}\right)$ according to Table 4;
(6) for a given $\kappa_{\omega }$, $\kappa_{u}$ and $\eta_{o}\left(\kappa_{u}\right)$, determine $M_{cr,o}\left(\kappa_{\omega },\kappa_{u},\kappa_{v}=0\right)$ using the Formula (7);
(7) for a given $\kappa_{\omega }$, determine $M_{cr}\left(\kappa_{\omega },\kappa_{u}=0,\kappa_{v}=1\right)$ using the Formula (8) and $M_{cr}\left(\kappa_{\omega },\kappa_{u}=1,\kappa_{v}=1\right)$ the Formula (9);
(8) for a given $\kappa_{u}$, determine the interaction coefficient $\eta_{u}\left(\kappa_{u}\right)$ according to Table 7;
(9) for a given $\kappa_{\omega }$, $\kappa_{u}$ and $\eta_{u}\left(\kappa_{u}\right)$, determine $M_{cr,u}\left(\kappa_{\omega },\kappa_{u},\kappa_{v}=1\right)$ using the Formula (10);
(10) for a given $\kappa_{\omega }$, $\kappa_{u}$ and $\kappa_{v}$, determine the interaction coefficient $\eta \left(\kappa_{v}\right)$ according to Table 1;
(11) for a given $M_{cr,o}\left(\kappa_{\omega },\kappa_{u},\kappa_{v}=0\right)$, $M_{cr,u}\left(\kappa_{\omega },\kappa_{u},\kappa_{v}=1\right)$ and $\eta \left(\kappa_{v}\right)$, estimate the elastic critical moment $M_{cr}\left(\kappa_{\omega },\kappa_{u},\kappa_{v}\right)$ using the Formula (5).

## 6. Results and Comparisons

Table 8 compares the results of Mcr calculations according to LTBeamN—col. V with the results of other authors: [37]—col. VI, [3]—col. VIII and calculations according to Formula (5)—col. X. As in [37] and [3], the results of FEM simulations—col. V were adopted as a reference point for comparison. A simply supported beam ($\kappa_{u}$ = 0, $\kappa_{v}$ = 0) elastically restrained against warping (0 < $\kappa_{\omega }$ < 1) analysed in [37] was assumed for the analysis (i.e., IPE 500, *I_z_* = 2141.7 cm^4^, *I_T_* = 89.006 cm^4^, *I_ω_* = 1,254,300 cm^6^, *L* = 8 m). The concentrated force load at the mid-span and the uniform load applied at the cross-section shear center were taken into account, which enabled the use of formulas derived in [3].

From the comparison of the results presented in Table 8 shows that the maximum differences between the analysed methods (approaches) in relation to FEM do not exceed: +0.8% according to [37]; +1.5% according to [3]; and +0.6% according to Formula (5), which is acceptable from the engineering point of view.

In a further detailed comparative analysis (covering the entire range of elastic restraints), the authors considered steel beams (*E* = 210 GPa, *G* = 81 GPa) made of IPE300, HEA300 and HEB300 sections with spans *L* = 5 and 7 m and IPE500, HEA500 and HEB500 sections with spans *L* = 8 and 10 m. Note: The geometrical characteristics of the sections in the analytical calculations and FEM simulations (LTBeamN) were adopted according to [45]. In the calculations, the authors considered the load diagrams shown in Table 1 (CFL, UDL, TDL). Transverse loads were applied at the height of the top flange (*z_g_* = +*h*/2, TF), at the centre of gravity (*z_g_* = 0, CG) and at the height of the bottom flange (*z_g_* = −*h*/2, BF). Calculations were carried out for various mutually independent combinations of elastic fixity indexes: $\kappa_{i}$ = {0, 0.2, 0.4, 0.6, 0.8, 1}, where i=ω,u,v.

**Figure 5:**
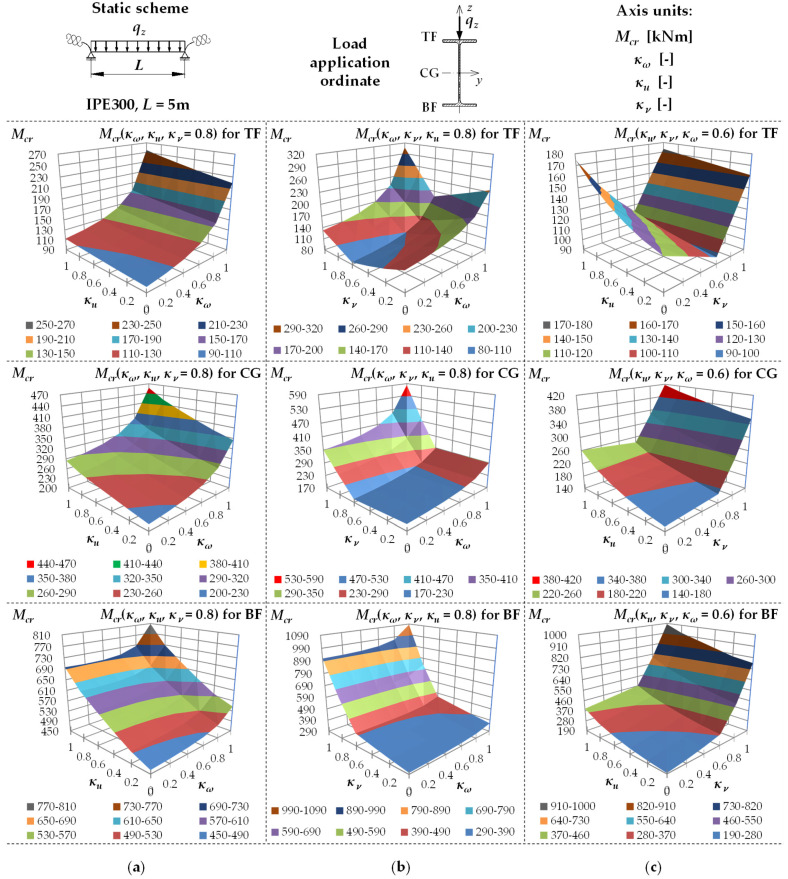
Trends in *M_cr_* variation according to Formula (5) as a function of the fixity index: (**a**) $\kappa_{\omega }$, $\kappa_{u}$ for $\kappa_{v}$ = 0.8, (**b**) $\kappa_{\omega }$, $\kappa_{v}$ for $\kappa_{u}$ = 0.8, (**c**) $\kappa_{u}$, $\kappa_{v}$ for $\kappa_{\omega }$ = 0.6, for beam under the uniform distributed load (UDL).

Figure 5 shows example variation surfaces of *M_cr_* as a function of $\kappa_{\omega }$, $\kappa_{u}$ and $\kappa_{v}$. The diagrams were drawn for the IPE300 beam with the span *L* = 5 m, loaded uniformly (UDL), successively at TF, CG and BF. Figure 5a shows the chart of $M_{cr}\left(\kappa_{\omega },\kappa_{u}\right)$ for $\kappa_{v}$ = 0.8, Figure 5b shows the chart of $M_{cr}\left(\kappa_{\omega },\kappa_{v}\right)$ dla $\kappa_{u}$ = 0.8, and Figure 5c shows the chart of $M_{cr}\left(\kappa_{u},\kappa_{v}\right)$ for $\kappa_{\omega }$ = 0.6. The critical moments were estimated using the formula (5).

The analysis of the charts indicated, among other things, that as the index $\kappa_{\omega }$ increased (Figure 5a,b)—regardless of the value of $\kappa_{u}$ and $\kappa_{v}$—there was a non-linear increase in *M_cr_*. The increase in $\kappa_{u}$ (Figure 5a,c), in turn, regardless of the value of $\kappa_{\omega }$ and $\kappa_{v}$, results in an essentially linear or mildly non-linear increase in *M_cr_*. For the UDL load, for $\kappa_{v}$ = 0.6 (Figure 5b,c), there is a characteristic bend in the *M_cr_* variation surface resulting from the change in the location of *M_y,max_* depending on $\kappa_{v}$ [21].

**Figure 6:**
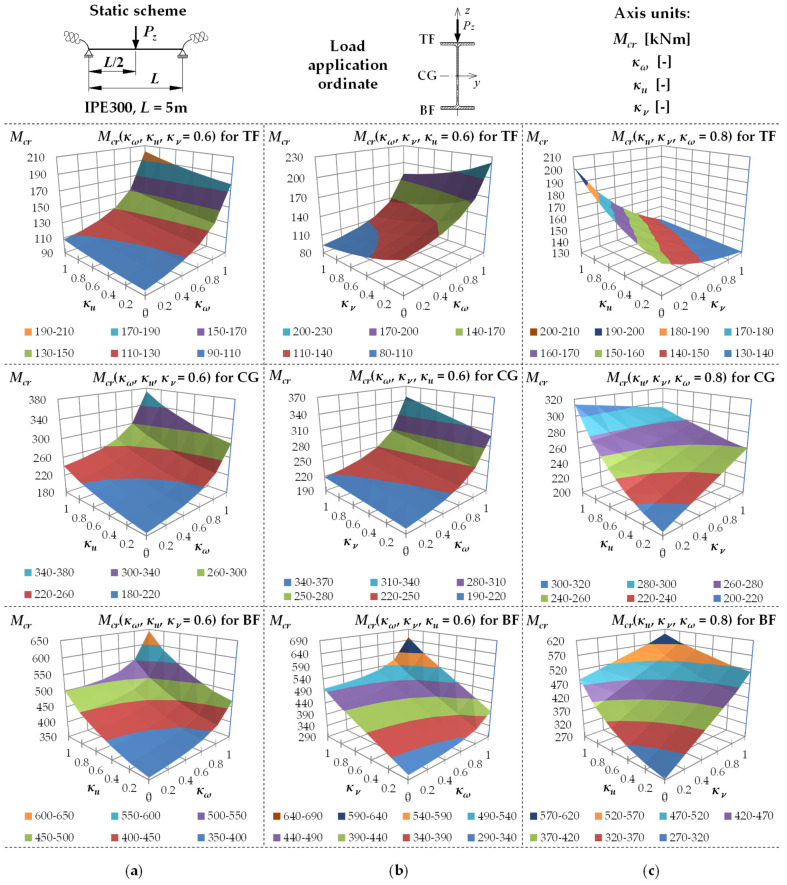
Trends in *M_cr_* variation according to Formula (5) as a function of the fixity index: (**a**) $\kappa_{\omega }$, $\kappa_{u}$ for $\kappa_{v}$ = 0.6, (**b**) $\kappa_{\omega }$, $\kappa_{v}$ for $\kappa_{u}$ = 0.6, (**c**) $\kappa_{u}$, $\kappa_{v}$ for $\kappa_{\omega }$ = 0.8, for beam under the concentrated force load (CFL).

Figure 6 shows example variation surfaces of *M_cr_* as a function of $\kappa_{\omega }$, $\kappa_{u}$ and $\kappa_{v}$ for the IPE300 beam, with the span *L* = 5 m, loaded with the concentrated load (CFL), successively at the height of TF, CG and BF. Figure 6a shows the chart of $M_{cr}\left(\kappa_{\omega },\kappa_{u}\right)$ for $\kappa_{v}$= 0.6, Figure 6b shows the chart of $M_{cr}\left(\kappa_{\omega },\kappa_{v}\right)$ for $\kappa_{u}$ = 0.6, and Figure 6c shows the chart of $M_{cr}\left(\kappa_{u},\kappa_{v}\right)$ for $\kappa_{\omega }$ = 0.8. The critical moments were estimated using the Formula (5).

The analysis of the charts indicated, among other things, that for CFL, the impact of $\kappa_{\omega }$ (Figure 6a,b) and $\kappa_{u}$ (Figure 6a,c) on the *M_cr_* variation was (as for UDL) more ($\kappa_{\omega }$) or less ($\kappa_{u}$) non-linear. The variation $M_{cr}\left(\kappa_{v}\right)$ (Figure 6b,c), in turn, was ‘smooth’, and in this case, the bend of the chart surface characteristic to UDL did not appear (Figure 5b,c). In the CFL case, the situation where the absolute values of the span and support moments *M_y_* are equal to each other does not occur until $\kappa_{v}=1$.

Table 9 shows examples of the results of calculations of $M_{cr}\left(\kappa_{\omega },\kappa_{u},\kappa_{v}\right)$ estimated using the Formula (5). The calculations were performed for: (1) different cross-section types (I, H), (2) different beam spans, (3) three load variants (CFL, UDL, TDL), 4) different combinations of elastic fixity indexes ($\kappa_{\omega }$, $\kappa_{u}$, $\kappa_{v}$). The load was applied at the height of the top flange (TF). The obtained results were compared with LTBeamN (FEM). Note: the results in Table 9 (columns VI, IX, XII) can be used to test the correct notation of the sequence of formulas according to Section 5, e.g., in a spreadsheet.

For the combinations of indexes ($\kappa_{\omega }$, $\kappa_{u}$, $\kappa_{v}$) adopted in Table 9 and the diagrams of the loads applied at the height of the top flange (TF), the *M_cr_* values estimated using the Formula (5) were different from FEM (LTBeamN), ranging from −7.2% (for a HEA300 beam spanning *L* = 7 m with TDL loading) to up to +3.4% (for the I400 beam spanning *L* = 9 m with UDL loading).

Table 10 compares the differences in results between the critical moments $M_{cr}\left(\kappa_{\omega },\kappa_{u},\kappa_{v}\right)$ estimated using the Formula (5) and those determined in the LTBeamN (FEM) software (v. 1.0.3). Detailed calculations were carried out for the IPE300, HEA300 and HEB300 beams with spans of *L* = 5 and 7 m and the IPE500, HEA500 and HEB500 beams with spans of *L* = 8 and 10 m, for the loading diagrams: CFL, UDL and TDL, applied successively at three different heights of the section (TF, CG, BF). The calculations were performed for different, mutually independent combinations of the values of $\kappa_{i}$ = {0, 0.2, 0.4, 0.6, 0.8, 1}, where i=ω,u,v. In total, more than 1600 simulations were performed. Column IV of Table 10 gives the extreme differences (Min ÷ Max), column V—the mean values (MV), column VI—the standard deviations (SD), and column VII—the coefficient of variation (CV in %).

For uniformly loaded beams (UDL) (Table 10, rows 4 to 6), the extreme percentage differences in the values of $M_{cr}\left(\kappa_{\omega },\kappa_{u},\kappa_{v}\right)$ estimated according to (5), in comparison with LTBeamN (FEM), ranged from −4.3% for TF to +5.3% for BF. For beams loaded with a concentrated load (CFL) or a non-uniform load (TDL), the discrepancies in the results were slightly higher than for UDL. For CFL (rows 1 to 3), the extreme differences were in the range of −6.9% (for BF, HEB500, *L* = 8 m, $\kappa_{\omega }$ = 0.6, $\kappa_{u}$ = 1, $\kappa_{v}$ = 0.6 and $\kappa_{\omega }$ = $\kappa_{u}$ = $\kappa_{v}$ = 0.8) to +8.0% (for BF, HEB300, *L* = 5 m, $\kappa_{\omega }$ = 0, $\kappa_{u}$ = 1, $\kappa_{v}$ = 1). For TDL (rows 7 to 9), in turn, the differences were in the range of −8.2% (for TF, HEB300, *L* = 5 m, $\kappa_{\omega }$ = 0.8, $\kappa_{u}$ = 1, $\kappa_{v}$ = 0.8) to +7.2% (for TF, IPE300, *L* = 7 m, $\kappa_{\omega }$ = 0, $\kappa_{u}$ = 1, $\kappa_{v}$ = 1).

Taking into account the statistical parameters (MV, SD, CV) for individual groups (Table 10, rows 1 to 9) and for the entire population of calculated beams (Table 10, row 10), it can be concluded that the obtained solution gives sufficiently good results from the engineering point of view.

The observed trend in the extreme (Min ÷ Max) differences in the results between the estimation according to the Formula (5) and LTBeamN is that the largest differences were obtained for the combinations of values of ($\kappa_{\omega }$, $\kappa_{u}$, $\kappa_{v}$) where at least one of the indexes ($\kappa_{u}$ and/or $\kappa_{v}$) had a value of 1 (i.e., complete restraint was present). In the case of $\kappa_{\omega }$ = 1, in turn, the differences were significantly smaller.

The observed differences in the results are related to, among other things, to the following factors: (1) consideration of the interaction of three important parameters of the elastic restraint of the beam ($\kappa_{\omega }$, $\kappa_{u}$, $\kappa_{v}$) in a relatively simple approximation Formula (5), (2) simplified approximation of the interaction coefficient $\eta_{u}\left(\kappa_{u}\right)$ (Table 7) [38], (3) variation of the location of the maximum moment *M_y,max_* depending on $\kappa_{v}$ (for UDL and TDL loads) identified with the critical moment *M_cr_* [21] and (4) the asymmetric form of lateral torsional buckling in the case of asymmetric load distribution (TDL).

Table 11 presents selected results of the sensitivity of the critical moment, calculated using Formula (5), to possible errors in the estimation of elastic restraint indexes. Detailed calculations were performed, among others, for an HEA300 beam, *L* = 8 m, for the base values of the indexes $\kappa_{\omega }$ = $\kappa_{u}$ = $\kappa_{v}$ = 0.3, 0.5, 0.7, under concentrated force loading and uniform loading. The results presented refer to the simultaneous overestimation of the $\kappa_{\omega }$ and $\kappa_{u}$ indexes (corresponding to the boundary conditions of lateral torsional buckling) at a level of +5%.

In the case of simultaneous overestimation of the $\kappa_{\omega }$ and $\kappa_{u}$ indexes, their impact on the *M_cr_* value depends on the base value—as the indices indexes, the discrepancy in *M_cr_* increases. In none of the tested cases did the discrepancy exceed the assumed estimation error of $\kappa_{\omega }$ and $\kappa_{u}$ at +5%.

Of course, the condition for obtaining a sufficiently good estimate of *M_cr_* from Formula (5) is to determine the elastic restraint indexes as accurately as possible.

## 7. Calculation Example

*Problem:* For a steel (*E* = 210 GPa, *G* = 81 GPa) beam made of an IPE400 section (*I_z_* = 1320 cm^4^, *I_t_* = 52.4 cm^4^, *I_ω_* = 490,000 cm^6^ [45]) with span *L* = 7.5 m, uniformly loaded (UDL) at the height of the top flange (TF), estimate the elastic critical moment *M_cr_* according to the procedure described in Section 5.

*Data:* the parameters of the elastic restraint of the beam at the support nodes are, respectively: $\alpha_{\omega }$ = 58.31 kNm^3^/rad, $\alpha_{u}$ = 580.8 kNm, $\alpha_{\nu }$ = 66,614.4 kNm/rad.

Compare the results obtained with *M_cr_* calculated for the conventional ‘fork’ support (i.e., for $\alpha_{\omega }=\alpha_{u}=0$) and considering the longitudinal distribution of the moment *M_y_* due to stiffness $\alpha_{\nu }$.

Furthermore, compare the differences in the estimation of the ‘lower order’ *M_cr_* components compared to LTBeamN.

*Solution:*

(A) Elastically restrained beam:

The elastic fixity indexes determined from Formulas (1)–(3) were, respectively: $\kappa_{\omega }$ = 0.68 (1), $\kappa_{u}$ = 0.44 (2), $\kappa_{v}$ = 0.72 (3).

Calculation of *M_cr_* components of the lower orders:

$M_{cr}\left(\kappa_{\omega },\kappa_{u}=0,\kappa_{v}=0\right)$ = 181.44 kNm (8); $M_{cr}\left(\kappa_{\omega },\kappa_{u}=1,\kappa_{v}=0\right)$ = 269.02 kNm (9)

$\eta_{o}\left(\kappa_{u}\right)$ = 0.285 Table 4; $M_{cr,o}\left(\kappa_{\omega },\kappa_{u},\kappa_{v}=0\right)$ = 206.41 kNm (7)

$M_{cr}\left(\kappa_{\omega },\kappa_{u}=0,\kappa_{v}=1\right)$ = 258.13 kNm (8); $M_{cr}\left(\kappa_{\omega },\kappa_{u}=1,\kappa_{v}=1\right)$ = 280.35 kNm (9)

$\eta_{u}\left(\kappa_{u}\right)$ = 0.430 Table 7; $M_{cr,u}\left(\kappa_{\omega },\kappa_{u},\kappa_{v}=1\right)$ = 267.69 kNm (10)

$\eta \left(\kappa_{v}\right)$ = −0.209 Table 1

$M_{cr}\left(\kappa_{\omega },\kappa_{u},\kappa_{v}\right)$ **= 193.61 kNm** (5)

Verification: Critical moment according to the LTBeamN software (v. 1.0.3, FEM): 197.29 kNm (a difference of approximately −1.8%).

(B) Beam with ‘fork’ support (i.e., for $\alpha_{\omega }=\alpha_{u}=0$) considering the longitudinal distribution of *M_y_* for $\alpha_{v}=66,614.4\;kNm/rad$
  Critical moment according to the LTBeamN software (v. 1.0.3, FEM): *M_cr_* = 148.01 kNm.
  The percentage increase in *M_cr_* after considering the elastic restraint of the beam (*M_cr_* according to (5)) was +31%.

*Conclusion:* Considering the elastic restraint ($\alpha_{\omega },\;\alpha_{u},\;\alpha_{\nu }$) of the beam at the support nodes made it possible to increase the elastic critical moment *M_cr_* by +31% in comparison with the conventionally adopted “fork” support ($\alpha_{\omega }=\alpha_{u}=0$).

(C) Differences in the estimation of ‘lower order’ *M_cr_* components compared to LTBeamN:
  Table 12 shows a comparison of the ‘lower order’ critical moments estimated using the approximation Formulas: (7)–(10) with the values determined in the LTBeamN software (v. 1.0.3).

*Conclusion:* The approximation Formulas (8) and (9), as well as (7) and (10), allow for the correct estimation of the lower-order critical moments of lateral torsional buckling in comparison with the LTBeamN software (v. 1.0.3).

In the vast majority of cases, the obtained differences (min ÷ max) of *M_cr_* components (Table 12—lines 1 to 6) do not exceed the difference for the final value, i.e., −1.8%, (Table 12—line 7). The exception is the case of full restraint against lateral rotation ($\kappa_{u}$ = 1) and against rotation in the bending plane *M_y_* ($\kappa_{v}$ = 1), for which the discrepancy was +2.2%. As mentioned earlier in Chapter 6, the largest discrepancies in estimates were obtained for cases containing indexes $\kappa_{u}$ = $\kappa_{v}$ = 1. However, the obtained accuracy of the *M_cr_* estimate is acceptable from an engineering point of view.

## 8. Summary and Conclusions

Consideration of the actual conditions of spatial elastic restraint of the beam at the support nodes, i.e., the elastic restraint against: (1) warping ($\alpha_{\omega })$, (2) rotation in the lateral torsional buckling plane $(\alpha_{u}$) and (3) rotation in the main bending plane ($\alpha_{\nu })$, leads to a more accurate calculation of the critical moment of lateral torsional buckling. With this approach, the reduction factor for lateral torsional buckling and the design resistance of the beam can be determined more accurately, leading to a more optimal design of such members. However, in order to consider the potential reserves of the resistance to lateral torsional buckling resulting from the elastic properties of the nodes, it is necessary to precisely determine the relevant stiffnesses of restrain ($\alpha_{\omega }$, $\alpha_{u}$, $\alpha_{\nu }$), which depend on the properties of the nodes and on the properties of other members of the system that reach the nodes. Further research is therefore needed to determine the stiffness parameters of beam nodes incorporated in of flat and spatial structural systems. This will be researched further by the authors of this article.

It should also be noted here that in some structures with simple nodes (nominally pinned nodes), the support conditions of the beams may be even weaker than the ‘fork’ support, which should be considered in the design calculations.

In the present study, building on their previous research [20,21,36,38], the authors have proposed an approximation Formula (5) for the elastic critical moment of lateral torsional buckling of the beam, with consideration of the following at the support nodes: (1) elastic restraint against warping, (2) elastic restraint against lateral rotation about the minor axis of the cross-section and (3) elastic restraint against rotation about the major axis of the cross-section. In the formula for $M_{cr}\left(\kappa_{\omega },\kappa_{u},\kappa_{v}\right)$, the authors used the concept of an integrated interaction coefficient, which enabled the simultaneous and independent consideration of the full range of node stiffnesses, i.e., from simple support of the beam against bending *M_y_* ($\kappa_{v}=0$) and the ‘fork’ support against lateral torsional buckling ($\kappa_{\omega }=0$, $\kappa_{u}=0$) up to its complete restraint ($\kappa_{\omega }=1$, $\kappa_{u}=1$, $\kappa_{v}=1$). To the authors’ knowledge, a theoretical solution (exact, analytical or approximative) to the presented problem has not yet been offered in the literature. However, the numerical solution of individual cases can be obtained using the finite element method, e.g., according to LTBeamN.

Based on the results obtained in the work, it can be concluded that the critical moment of lateral torsional buckling determined from the Formula (5) as a function of three technically relevant indexes $M_{cr}\left(\kappa_{\omega },\kappa_{u},\kappa_{v}\right)$ give, in the great majority of cases, an estimate of *M_cr_* that is sufficient, from an engineering point of view, in comparison with the FEM. For all cases examined in the work (over 1600 simulations), the mean value of the ratio *M_cr_* according to Formula (5) to *M_cr_* according to FEM was 1.006, the standard deviation was 0.028, and the coefficient of variation was 2.8%. In most cases, the maximum differences in estimation should not exceed ±5% to ±8%, and in very rare cases—up to ±10% (no such discrepancy was recorded in the conducted simulations).

The error in the estimation of the coefficient of lateral torsional buckling ($\chi_{LT}$) for hot-rolled I-sections (with *M_cr_* estimated with an error of +5%) should not exceed a maximum of +2.3% for a range of relative slendernesses ${\bar{\lambda }}_{LT}$ from 0.5 to 1 and a maximum of +4.1% for ${\bar{\lambda }}_{LT}$ in the range from 1.5 to 3, which is technically acceptable. With an *M_cr_* estimation error of up to +10%, differences for $\chi_{LT}$ should not exceed, respectively: +4.5% for 0.5 <${\bar{\;\lambda }}_{LT}$ < 1 and +8.5% for 1.5 <${\bar{\;\lambda }}_{LT}$ < 3. Achieving greater estimation accuracy $M_{cr}\left(\kappa_{\omega },\kappa_{u},\kappa_{v}\right)$ leads to a significant extension of the approximation formulas.

The approach presented in this paper can be used both for the initial selection of the section as well as (in the three cases of load distribution and three typical points of load application—TF, CG, BF) for main design work. Above all, it allows *M_cr_* calculations to be verified with FEM software (e.g., LTBeamN). A computational test using approximation formulas for a simple load distribution (e.g., UDL) and the defined elastic restraints, confirming the result of FEM simulations could confirm the validity of the elastic restraint model. Once the support model is verified in this way, it will be possible to return to the FEM simulation by specifying the load scheme required in the design. Such a check can be very useful especially for less experienced designers and can be used for the necessary correction of, for example, incorrect notation of beam boundary conditions in FEM software.

This approach, i.e., confirming important design parameters, in this case, the critical moment of lateral torsional buckling, with results from two sources (e.g., FEM and approximation formulas) increases the safety of the structure already at the design stage and should be the standard approach.

In practical calculations $M_{cr}\left(\kappa_{\omega },\kappa_{u},\kappa_{v}\right)$ using the approximation formulas and the integrated interaction coefficient, it should be assumed that the calculations are carried out in the $\kappa_{v}$ interval of 0.1 to 0.9, which covers most technically relevant cases of the elastic restraint of beams. In the extreme ranges, i.e., from 0 to 0.1 and from 0.9 to 1, the *M_cr_* values should be linearly interpolated, as described in Section 3.2.

In addition, the derived formulas can be used to conduct a series of analyses of the interaction influence of individual design parameters, including the influence of elastic support restraints, on *M_cr_*. The formulas are suitable for use in spreadsheets and allow for the calculation process to be automated. Performing such an analysis using FEM would involve a very large number of separate simulations. Examples of such analyses will be the subject of further research by the authors.

The consideration of the actual boundary conditions at beam support nodes, in comparison with the ‘fork’ support alternatively adopted in design practice, is an important development in contemporary methods for designing steel structures. The aim is to create structural safety not on the basis of unknown reserves of resistance (e.g., between the ‘fork’ support model and the elastic restraint existing in real conditions), but on objective reliability criteria.

## Figures and Tables

**Table 1:** Coefficients of interaction $\eta \left(\kappa_{v}\right)$ for $M_{cr}\left(\kappa_{\omega },\kappa_{u},\kappa_{v}\right)$.

Item	Static Scheme	Coefficients		
I	II	III		
1	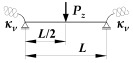	TF [21]	0 < *κ_v_* < 1	$\eta \left(\kappa_{v}\right)=\frac{2\kappa_{v}}{1+\kappa_{v}}$
		CG [21]	0 < *κ_v_* < 1	$\eta \left(\kappa_{v}\right)=\kappa_{v}$
		BF [21]	0 < *κ_v_* < 1	$\eta \left(\kappa_{v}\right)=0.95\kappa_{v}$
2	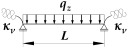	TF [21]	0 < *κ_v_* ≤ 0.6	$\eta \left(\kappa_{v}\right)=\frac{\kappa_{v}\left[\left(2.333-0.333\kappa_{v}\right)M_{o}+\left(0.5\kappa_{v}-1.5\right)M_{u}\right]}{\left(1+\kappa_{v}\right)\left(M_{o}-M_{u}\right)}$
			0.6 < *κ_v_* < 1	$\eta \left(\kappa_{v}\right)=\frac{\left[1+\kappa_{v}\left(1.333\kappa_{v}-0.333\right)\right]M_{o}-2\kappa_{v}^{2}M_{u}}{\left(1+\kappa_{v}\right)\left(M_{o}-M_{u}\right)}$
		CG	0 < *κ_v_* ≤ 0.6	$\eta \left(\kappa_{v}\right)=Y\cdot \kappa_{v}+Z$ $Y=\left(0.359\kappa_{\omega }-0.705\right)\kappa_{u}^{2}-\left(0.118\kappa_{\omega }-0.232\right)\kappa_{u}-\left(0.01\kappa_{\omega }-0.118\right)$ $Z=-\left(0.017\kappa_{\omega }-0.008\right)\kappa_{u}^{2}+\left(0.017\kappa_{\omega }-0.004\right)\kappa_{u}$
			0.6 < *κ_v_* < 1	$\eta \left(\kappa_{v}\right)=X\cdot \kappa_{\nu }^{2}+Y\cdot \kappa_{\nu }+Z$ $X=-\left(0.495\kappa_{\omega }+0.13\right)\kappa_{u}^{2}+\left(0.688\kappa_{\omega }-0.68\right)\kappa_{u}+\left(0.216\kappa_{\omega }+1.017\right)$ $Y=\left(0.269\kappa_{\omega }+1.259\right)\kappa_{u}^{2}-\left(0.94\kappa_{\omega }-0.749\right)\kappa_{u}-\left(0.33\kappa_{\omega }-0.696\right)$ $Z=\left(0.227\kappa_{\omega }-1.129\right)\kappa_{u}^{2}+\left(0.252\kappa_{\omega }-0.069\right)\kappa_{u}+\left(0.114\kappa_{\omega }-0.713\right)$
		BF	0 < *κ_v_* ≤ 0.6	$\eta \left(\kappa_{v}\right)=X\cdot \kappa_{\nu }^{2}+Y\cdot \kappa_{\nu }$ $X=\left(0.464\kappa_{\omega }-0.553\right)\kappa_{u}^{3}-\left(0.353\kappa_{\omega }-0.661\right)\kappa_{u}^{2}-\left(0.014\kappa_{\omega }+0.301\right)\kappa_{u}-\left(0.04\kappa_{\omega }-0.169\right)$ $Y=-\left(0.236\kappa_{\omega }-0.226\right)\kappa_{u}^{3}+\left(0.182\kappa_{\omega }-0.391\right)\kappa_{u}^{2}+\left(0.015\kappa_{\omega }+0.222\right)\kappa_{u}-\left(0.019\kappa_{\omega }-0.175\right)$
			0.6 < *κ_v_* < 1	$\eta \left(\kappa_{v}\right)=X\cdot \kappa_{\nu }^{2}+Y\cdot \kappa_{\nu }+Z$ $X=-\left(0.428\kappa_{\omega }-0.185\right)\kappa_{u}^{2}+\left(0.945\kappa_{\omega }-1.422\right)\kappa_{u}-\left(0.079\kappa_{\omega }-2.072\right)$ $Y=\left(0.647\kappa_{\omega }-0.068\right)\kappa_{u}^{2}-\left(1.501\kappa_{\omega }-2.129\right)\kappa_{u}+\left(0.19\kappa_{\omega }-1.228\right)$ $Z=-\left(0.219\kappa_{\omega }+0.117\right)\kappa_{u}^{2}+\left(0.556\kappa_{\omega }-0.707\right)\kappa_{u}-\left(0.111\kappa_{\omega }-0.156\right)$
3	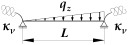	TF [21]	0 < *κ_v_* ≤ 0.564	$\eta \left(\kappa_{v}\right)=\frac{\kappa_{v}\left[\left(2.386-0.386\kappa_{v}\right)M_{o}+\left(0.495\kappa_{v}-1.283\right)M_{u}\right]}{\left(1+\kappa_{v}\right)\left(M_{o}-M_{u}\right)}$
			0.564 < *κ_v_* < 1	$\eta \left(\kappa_{v}\right)=\frac{\left[1+\kappa_{v}\left(1.386\kappa_{v}-0.386\right)\right]M_{o}-1.778\kappa_{v}^{2}M_{u}}{\left(1+\kappa_{v}\right)\left(M_{o}-M_{u}\right)}$
		CG	0 < *κ_v_* ≤ 0.564	$\eta \left(\kappa_{v}\right)=Y\cdot \kappa_{v}+Z$ $Y=\left(0.218\kappa_{\omega }-0.46\right)\kappa_{u}^{2}-\left(0.047\kappa_{\omega }-0.134\right)\kappa_{u}-\left(0.017\kappa_{\omega }-0.12\right)$ $Z=-\left(0.005\kappa_{\omega }+0.01\right)\kappa_{u}^{2}+\left(0.01\kappa_{\omega }+0.004\right)\kappa_{u}$
			0.564 < *κ_v_* < 1	$\eta \left(\kappa_{v}\right)=X\cdot \kappa_{\nu }^{2}+Y\cdot \kappa_{\nu }+Z$ $X=-\left(0.761\kappa_{\omega }+0.097\right)\kappa_{u}^{2}+\left(0.457\kappa_{\omega }-0.225\right)\kappa_{u}+\left(0.322\kappa_{\omega }+1.137\right)$ $Y=\left(0.901\kappa_{\omega }+0.783\right)\kappa_{u}^{2}-\left(0.662\kappa_{\omega }-0.17\right)\kappa_{u}-\left(0.48\kappa_{\omega }-0.349\right)$ $Z=-\left(0.14\kappa_{\omega }+0.686\right)\kappa_{u}^{2}+\left(0.205\kappa_{\omega }+0.055\right)\kappa_{u}+\left(0.158\kappa_{\omega }-0.486\right)$
		BF	0 < *κ_v_* ≤ 0.564	$\eta \left(\kappa_{v}\right)=X\cdot \kappa_{\nu }^{2}+Y\cdot \kappa_{\nu }$ $X=\left(0.395\kappa_{\omega }-0.852\right)\kappa_{u}^{3}-\left(0.448\kappa_{\omega }-1.449\right)\kappa_{u}^{2}+\left(0.006\kappa_{\omega }-0.602\right)\kappa_{u}-\left(0.031\kappa_{\omega }-0.163\right)$ $Y=-\left(0.195\kappa_{\omega }-0.479\right)\kappa_{u}^{3}+\left(0.255\kappa_{\omega }-0.966\right)\kappa_{u}^{2}+\left(0.014\kappa_{\omega }+0.423\right)\kappa_{u}-\left(0.021\kappa_{\omega }-0.14\right)$
			0.564 < *κ_v_* < 1	$\eta \left(\kappa_{v}\right)=X\cdot \kappa_{\nu }^{2}+Y\cdot \kappa_{\nu }+Z$ $X=-\left(0.557\kappa_{\omega }+0.363\right)\kappa_{u}^{2}+\left(0.434\kappa_{\omega }-0.349\right)\kappa_{u}+\left(0.023\kappa_{\omega }+2.209\right)$ $Y=\left(0.818\kappa_{\omega }+0.772\right)\kappa_{u}^{2}-\left(0.691\kappa_{\omega }-0.434\right)\kappa_{u}+\left(0.014\kappa_{\omega }-1.464\right)$ $Z=-\left(0.261\kappa_{\omega }+0.408\right)\kappa_{u}^{2}+\left(0.256\kappa_{\omega }-0.086\right)\kappa_{u}-\left(0.036\kappa_{\omega }-0.255\right)$

TF—load applied to the top flange (see Figure 4); CG—load applied to the centre of gravity of the cross section (see Figure 4); BF—load applied to the bottom flange (see Figure 4); $M_{o}=M_{cr,o}\left(\kappa_{\omega },\kappa_{u},\kappa_{v}=0\right)$—the critical moment for a simply supported for *M_y_* $\left(\kappa_{v}=0\right)$ beam; $M_{u}=M_{cr,u}\left(\kappa_{\omega },\kappa_{u}{,\kappa }_{v}=1\right)$—the critical moment for a bilaterally fixed for *M_y_* $\left(\kappa_{v}=1\right)$ beam.

**Table 2:** Data from [36]. Coefficients $B_{1},\;B_{2},\;B_{3},\;B_{4}$ for $M_{cr}\left(\kappa_{\omega },\kappa_{u}=0,\kappa_{v}=0\right)$.

Item	Static Scheme	Coefficients
I	II	III
1	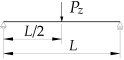	$B_{1}=7.242\cdot \left(1.563-2.5\kappa_{\omega }+\kappa_{\omega }^{2}\right)$ $B_{2}=1.522-2.467\kappa_{\omega }+\kappa_{\omega }^{2}$ $B_{3}=19.248\cdot B_{2}\cdot (1.457-2.4\kappa_{\omega }+\kappa_{\omega }^{2})$ $B_{4}=231.816\cdot B_{2}\cdot (1.2-\kappa_{\omega })$
2	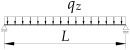	$B_{1}=5.25\cdot (1.476-2.429\kappa_{\omega }+\kappa_{\omega }^{2})$ $B_{2}=1.507-2.455\kappa_{\omega }+\kappa_{\omega }^{2}$ $B_{3}=13.092\cdot B_{2}\cdot (1.457-2.4\kappa_{\omega }+\kappa_{\omega }^{2})$ $B_{4}=157.633\cdot B_{2}\cdot (1.2-\kappa_{\omega })$
3	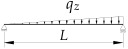	$B_{1}=5.322\cdot (1.476-2.429\kappa_{\omega }+\kappa_{\omega }^{2})$ $B_{2}=1.507-2.455\kappa_{\omega }+\kappa_{\omega }^{2}$ $B_{3}=13.624\cdot B_{2}\cdot (1.457-2.4\kappa_{\omega }+\kappa_{\omega }^{2})$ $B_{4}=163.486\cdot B_{2}\cdot (1.2-\kappa_{\omega })$

**Table 3:** Data from [38]. Coefficients $B_{1},\;B_{2},\;B_{3},\;B_{4},\;D_{1}$ for $M_{cr}\left(\kappa_{\omega },\kappa_{u}=1,\kappa_{v}=0\right)$.

Item	Static Scheme	Coefficients
I	II	III
1	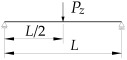	$B_{1}=22.5\cdot \left(1.563-2.5\kappa_{\omega }+\kappa_{\omega }^{2}\right)$ $B_{2}=1.554-2.493\kappa_{\omega }+\kappa_{\omega }^{2}$ $B_{3}=60\cdot B_{2}\cdot (1.457-2.4\kappa_{\omega }+\kappa_{\omega }^{2})$ $B_{4}=720\cdot B_{2}\cdot (1.2-\kappa_{\omega })$ $D_{1}=0.92+0.07\cdot \frac{z_{g}}{h}-0.03\kappa_{\omega }$
2	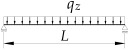	$B_{1}=18.375\cdot (1.476-2.429\kappa_{\omega }+\kappa_{\omega }^{2})$ $B_{2}=1.563-2.5\kappa_{\omega }+\kappa_{\omega }^{2}$ $B_{3}=45.937\cdot B_{2}\cdot (1.457-2.4\kappa_{\omega }+\kappa_{\omega }^{2})$ $B_{4}=551.25\cdot B_{2}\cdot (1.2-\kappa_{\omega })$ $D_{1}=0.96+0.07\cdot \frac{z_{g}}{h}-0.03\kappa_{\omega }$
3	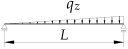	$B_{1}=18.816\cdot (1.476-2.429\kappa_{\omega }+\kappa_{\omega }^{2})$ $B_{2}=1.563-2.5\kappa_{\omega }+\kappa_{\omega }^{2}$ $B_{3}=48.169\cdot B_{2}\cdot (1.457-2.4\kappa_{\omega }+\kappa_{\omega }^{2})$ $B_{4}=578.028\cdot B_{2}\cdot (1.2-\kappa_{\omega })$ $D_{1}=0.96+0.07\cdot \frac{z_{g}}{h}-0.03\kappa_{\omega }$

**Table 4:** Data from [38]. Coefficients of interaction $\eta_{o}\left(\kappa_{u}\right)$ for $M_{cr,o}\left(\kappa_{\omega },\kappa_{u},\kappa_{v}=0\right)$.

Item	Static Scheme	Coefficients
I	II	III
1	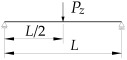	$\eta_{o}\left(\kappa_{u}\right)=\left(0.66-0.17\frac{z_{g}}{h}\right)\kappa_{u}^{2}+\left(0.27+0.25\frac{z_{g}}{h}\right)\kappa_{u}-0.02\frac{z_{g}}{h}+0.01$
2	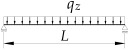	
3	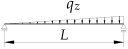	

**Table 5:** Data from [21]. Coefficients $B_{1},\;B_{2},\;B_{3},\;B_{4}$ for $M_{cr}\left(\kappa_{\omega },\kappa_{u}=0,\kappa_{v}=1\right)$.

Item	Static Scheme	Coefficients
I	II	III
1	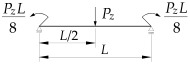	$B_{1}=23.333\cdot \left(1.563-2.5\kappa_{\omega }+\kappa_{\omega }^{2}\right)$ $B_{2}=1.522-2.467\kappa_{\omega }+\kappa_{\omega }^{2}$ $B_{3}=31.032\cdot B_{2}\cdot (1.457-2.4\kappa_{\omega }+\kappa_{\omega }^{2})$ $B_{4}=372.934\cdot B_{2}\cdot (1.2-\kappa_{\omega })$
2	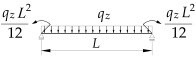	$B_{1}=42\cdot (1.476-2.429\kappa_{\omega }+\kappa_{\omega }^{2})$ $B_{2}=1.507-2.455\kappa_{\omega }+\kappa_{\omega }^{2}$ $B_{3}=69.692\cdot B_{2}\cdot (1.457-2.4\kappa_{\omega }+\kappa_{\omega }^{2})$ $B_{4}=839.664\cdot B_{2}\cdot (1.2-\kappa_{\omega })$
3	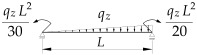	$B_{1}=49.033\cdot (1.476-2.429\kappa_{\omega }+\kappa_{\omega }^{2})$ $B_{2}=1.507-2.455\kappa_{\omega }+\kappa_{\omega }^{2}$ $B_{3}=102.445\cdot B_{2}\cdot (1.457-2.4\kappa_{\omega }+\kappa_{\omega }^{2})$ $B_{4}=1234.274\cdot B_{2}\cdot (1.2-\kappa_{\omega })$

**Table 6:** Data from [38]. Coefficients $B_{1},\;B_{2},\;B_{3},\;B_{4},\;D_{1}$ for $M_{cr}\left(\kappa_{\omega },\kappa_{u}=1,\kappa_{v}=1\right)$.

Item	Static Scheme	Coefficients
I	II	III
1	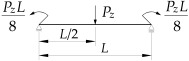	$B_{1}=45\cdot \left(1.563-2.5\kappa_{\omega }+\kappa_{\omega }^{2}\right)$ $B_{2}=1.458-2.415\kappa_{\omega }+\kappa_{\omega }^{2}$ $B_{3}=60\cdot B_{2}\cdot (1.457-2.4\kappa_{\omega }+\kappa_{\omega }^{2})$ $B_{4}=720\cdot B_{2}\cdot (1.2-\kappa_{\omega })$ $D_{1}=0.8+0.3\cdot \frac{z_{g}}{h}-0.05\kappa_{\omega }$
2	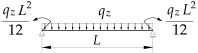	$B_{1}=70.56\cdot (1.476-2.429\kappa_{\omega }+\kappa_{\omega }^{2})$ $B_{2}=1.44-2.4\kappa_{\omega }+\kappa_{\omega }^{2}$ $B_{3}=117.6\cdot B_{2}\cdot (1.457-2.4\kappa_{\omega }+\kappa_{\omega }^{2})$ $B_{4}=1411.2\cdot B_{2}\cdot (1.2-\kappa_{\omega })$ $D_{1}=0.9+0.22\cdot \frac{z_{g}}{h}-0.05\kappa_{\omega }$
3	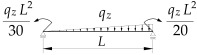	$B_{1}=84.672\cdot (1.476-2.429\kappa_{\omega }+\kappa_{\omega }^{2})$ $B_{2}=1.44-2.4\kappa_{\omega }+\kappa_{\omega }^{2}$ $B_{3}=169.344\cdot B_{2}\cdot (1.457-2.4\kappa_{\omega }+\kappa_{\omega }^{2})$ $B_{4}=2032.128\cdot B_{2}\cdot (1.2-\kappa_{\omega })$ $D_{1}=0.9+0.22\cdot \frac{z_{g}}{h}-0.05\kappa_{\omega }$

**Table 7:** Data from [38]. Coefficients of interaction $\eta_{u}\left(\kappa_{u}\right)$ for $M_{cr,u}\left(\kappa_{\omega },\kappa_{u},\kappa_{v}=1\right)$.

Item	Static Scheme	Coefficients
I	II	III
1	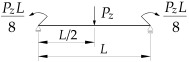	$\eta_{u}\left(\kappa_{u}\right)=\kappa_{u}+0.08\frac{z_{g}}{h}-0.05$
2	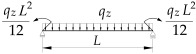	
3	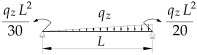	

**Table 8:** Comparison of the results of FEM simulations (LTBeamN) and calculations according to Formula (5) with the results of other authors [3,37] for $M_{cr}\left(\kappa_{\omega },\kappa_{u}=0,\kappa_{v}=0\right)$.

Item	Static Scheme	$\kappa_{\omega }$[-]	Ordinate of Load Application	$M_{cr}\left(\kappa_{\omega },\kappa_{u}=0,\kappa_{v}=0\right)$ [kNm]						
				LTBeamN [37]	Acc. to [37]	VI/V [-]	Acc. to [3]	VIII/V [-]	Formula (5) [kNm]	X/V [-]
I	II	III	IV	V	VI	VII	VIII	IX	X	XI
1	** 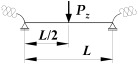 **	0	CG	380.89	380.69	0.999	381.92	1.003	382.38	1.004
2		0.25		396.10	396.50	1.001	396.61	1.001	398.09	1.005
3		0.5		421.19	421.51	1.001	421.51	1.001	423.05	1.004
4		0.75		467.05	468.68	1.003	470.16	1.007	469.23	1.005
5		1		584.79	588.86	1.007	591.35	1.011	587.26	1.004
6	** 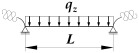 **	0	CG	316.26	316.24	1.000	316.66	1.001	316.91	1.002
7		0.25		329.72	329.80	1.000	329.70	1.000	330.35	1.002
8		0.5		351.15	351.36	1.001	351.77	1.002	351.83	1.002
9		0.75		391.05	392.35	1.003	394.91	1.010	392.01	1.002
10		1		495.20	499.06	1.008	502.87	1.015	498.06	1.006

**Table 9:** Comparison of $M_{cr}\left(\kappa_{\omega },\kappa_{u},\kappa_{v}\right)$.

Item	Section (*L* [m])	$\kappa_{\omega }$ [-]	$\kappa_{u}$ [-]	$\kappa_{v}$ [-]	Static Scheme Load Ordinate (*P_z_*, *q_z_*)—Top Flange (TF) of the Beam Cross-Section								
					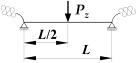			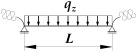			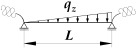		
					$M_{cr}\left(\kappa_{\omega },\kappa_{u},\kappa_{v}\right)$ [kNm]			$M_{cr}\left(\kappa_{\omega },\kappa_{u},\kappa_{v}\right)$ [kNm]			$M_{cr}\left(\kappa_{\omega },\kappa_{u},\kappa_{v}\right)$ [kNm]		
					Formula (5)	LTBeamN	%	Formula (5)	LTBeamN	%	Formula (5)	LTBeamN	%
							VI–VII			IX–X			XII–XIII
I	II	III	IV	V	VI	VII	VIII	IX	X	XI	XII	XIII	XIV
1	IPE 300 (7)	0.2	0.2	0.2	78.45	80.10	−2.1	68.42	69.67	−1.8	69.85	70.82	−1.4
2	IPE 500 (10)				218.23	223.08	−2.2	190.34	194.19	−2.0	194.30	197.42	−1.6
3	HEB 300 (5)				1317.96	1362.20	−3.2	1153.08	1197.90	−3.7	1177.27	1221.70	−3.6
4	HEA 500 (8)				1142.67	1176.70	−2.9	998.58	1035.00	−3.5	1019.53	1053.20	−3.2
5	IPE 300 (5)	0.2	0.4	0.8	99.83	99.96	−0.1	110.32	112.46	−1.9	119.91	128.56	−6.7
6	IPE 500 (8)				247.69	247.73	0.0	274.07	278.93	−1.7	297.80	318.73	−6.6
7	HEB 300 (7)				790.66	789.43	0.2	875.12	889.59	−1.6	950.83	1016.60	−6.5
8	HEA 500 (10)				795.04	793.14	0.2	880.57	894.56	−1.6	956.60	1021.80	−6.4
9	IPE 300 (7)	0.8	0.6	0.2	102.22	104.72	−2.4	95.16	96.07	−0.9	96.69	97.57	−0.9
10	IPE 500 (10)				287.68	294.75	−2.4	267.95	270.90	−1.1	272.28	275.16	−1.0
11	HEB 500 (8)				2243.47	2303.60	−2.6	2097.33	2135.00	−1.8	2131.28	2169.10	−1.7
12	HEA 300 (5)				1347.86	1383.70	−2.6	1266.66	1299.40	−2.5	1287.15	1326.70	−3.0
13	IPE 300 (5)	0.8	0.8	0.8	140.70	141.17	−0.3	169.25	171.05	−1.1	181.53	195.41	−7.1
14	IPE 500 (8)				341.18	342.02	−0.2	410.32	413.95	−0.9	440.06	472.50	−6.9
15	HEB 500 (10)				1490.13	1487.40	0.2	1791.04	1798.30	−0.4	1920.40	2049.60	−6.3
16	HEA 300 (7)				677.72	681.84	−0.6	815.48	825.99	−1.3	874.75	942.77	−7.2
17	HEB 800 (16)	0.6	0.2	0.4	1200.60	1230.70	−2.4	1039.30	1049.40	−1.0	1053.94	1066.40	−1.2
18	I 400 (9)				271.83	272.62	−0.3	236.82	228.99	+3.4	239.62	232.52	+3.1
19	HEA 700 (14)				928.41	955.87	−2.9	802.63	819.56	−2.1	814.36	833.09	−2.2
20	IPE 180 (5)				22.51	22.92	−1.8	19.52	19.46	+0.3	19.78	19.76	+0.1

**Table 10:** Comparison between analytical (Formula (5)) and numerical (LTBeamN) results of $M_{cr}\left(\kappa_{\omega },\kappa_{u},\kappa_{v}\right)$.

Item	Static Scheme	$\mathrm{Differences}\;\mathrm{in}\;M_{cr}\left(\kappa_{\omega },\kappa_{u},\kappa_{v}\right)$				
		Formula (5) vs. LTBeamN (FEM)				
		Ordinate of Load Application	$\mathrm{Min}\;÷\;\mathrm{Max}\;\left(\frac{\left(5\right)}{FEM}\right)$	$\mathrm{MV}\;\left(\frac{\left(5\right)}{FEM}\right)$	SD	CV [%]
I	II	III	IV	V	VI	VII
1	** 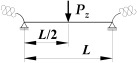 **	TF, *z_g_* = +*h*/2	0.968 ÷ 1.068	1.003	0.0223	2.2
2		CG, *z_g_* = 0	0.976 ÷ 1.043	1.004	0.0157	1.6
3		BF, *z_g_* = −*h*/2	0.931 ÷ 1.080	0.989	0.0301	3.0
4	** 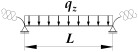 **	TF, *z_g_* = +*h*/2	0.957 ÷ 1.051	1.003	0.0234	2.3
5		CG, *z_g_* = 0	0.992 ÷ 1.034	1.013	0.0109	1.1
6		BF, *z_g_* = −*h*/2	0.975 ÷ 1.053	1.012	0.0165	1.6
7	** 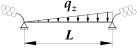 **	TF, *z_g_* = +*h*/2	0.918 ÷ 1.072	0.982	0.0481	4.9
8		CG, *z_g_* = 0	0.992 ÷ 1.070	1.027	0.0138	1.3
9		BF, *z_g_* = −*h*/2	0.984 ÷ 1.068	1.024	0.0177	1.7
10	All cases	TF, CG, BF	0.918 ÷ 1.080	1.006	0.0280	2.8

**Table 11:** Differences in *M_cr_* values (in %) when the $\kappa_{\omega }$ and $\kappa_{u}$ indices are revalued by +5%.

Item	Static Scheme	Ordinate of Load Application	$\kappa_{v}$ = 0.3 $\kappa_{\omega }$$\;=\;\kappa_{u}$ = 0.3 +5%	$\kappa_{v}$ = 0.5 $\kappa_{\omega }$$\;=\;\kappa_{u}$ = 0.5 +5%	$\kappa_{v}$ = 0.7 $\kappa_{\omega }$$\;=\;\kappa_{u}$ = 0.7 +5%
I	II	III	IV	V	VI
1	** 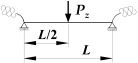 **	TF	+0.67	+1.43	+2.97
2		CG	+0.73	+1.43	+2.64
3		BF	+0.70	+1.27	+2.09
4	** 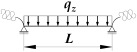 **	TF	+0.82	+1.77	+3.77
5		CG	+0.86	+1.65	+2.93
6		BF	+0.92	+1.59	+2.14

**Table 12:** Accuracy of estimation of ‘lower order’ *M_cr_* components.

Item	Elastic Fixity Indexes	*M_cr_* acc. LTBeamN (FEM)	Approximation Formulas		%
			*M_cr_* [kNm]	Formula	
I	II	III	IV	V	VI
1	$\kappa_{\omega }=0.68,\kappa_{u}=0,\kappa_{v}=0$	181.24	181.44	(8)	+0.1
2	$\kappa_{\omega }=0.68,\kappa_{u}=1,\kappa_{v}=0$	267.36	269.02	(9)	+0.6
3	$\kappa_{\omega }=0.68,\kappa_{u}=0.44,\kappa_{v}=0$	206.80	206.41	(7)	−0.2
4	$\kappa_{\omega }=0.68,\kappa_{u}=0,\kappa_{v}=1$	257.99	258.13	(8)	+0.1
5	$\kappa_{\omega }=0.68,\kappa_{u}=1,\kappa_{v}=1$	274.35	280.35	(9)	+2.2
6	$\kappa_{\omega }=0.68,\kappa_{u}=0.44,\kappa_{v}=1$	264.74	267.69	(10)	+1.1
7	$\kappa_{\omega }=0.68,\kappa_{u}=0.44,\kappa_{v}=0.72$	197.29	193.61	(5)	−1.8

## Data Availability

The original contributions presented in the study are included in the article. Further inquiries can be directed to the corresponding author.
